# Therapeutic evaluation of *Martynia annua* derived carbon dots in epileptic *Drosophila* model

**DOI:** 10.1038/s41598-025-07780-2

**Published:** 2025-07-01

**Authors:** Megha B. Abbigeri, Muskan Khan, Bothe Thokchom, Sapam Riches Singh, Santosh Mallikarjun Bhavi, Sudheendra Rao Kulkarni, B. P. Harini, G. Vidya Sagar Reddy, Chandramohan Govindasamy, Khalid M. Almutairi, Yarajarla Ramesh Babu

**Affiliations:** 1 Drosophila and Nanoscience Research Laboratory, Department of Applied Genetics, Karnatak University, Dharwad, Karnataka 580003 India; 2 Department of Zoology, Bangalore University, Bengaluru, Karnataka India; 3 Department of Biotechnology, Vikrama Simhapuri University, Nellore, Andhra Pradesh India; 4 Department of Community Health Sciences, College of Applied Medical Sciences, King Saud University, Riyadh, Saudi Arabia

**Keywords:** *Martynia annua*, Carbon dots, Carbamazepine, Epilepsy, Para bang senseless, Neuroscience, Biomaterials

## Abstract

This study investigates the synthesis and characterization of Carbon dots (MA-CDs) derived from the aqueous extract of *Martynia annua* and examining their potential effects in an epilepsy model *Drosophila melanogaster*. Phytochemical analysis confirmed the presence of saponin, terpeniods, and flavanoids in the leaf extract, which facilitated the green synthesis of MA-CDs. Physicochemical characterization revealed an absorbance peak at 326 nm, the mean size of the particle was 3.17 ± 0.16 nm, and moderate stability (−1.6 mV). To assess the therapeutic potential of MA-CDs alongside the antiepileptic drug Carbamazepine (CBZ), we conducted behavioral and cognitive assays in para bang senseless (para^bss1^) mutants of *Drosophila*, a model organism for epilepsy. Seizures induced by vortex and heat shock were significantly mitigated in a dose-dependent manner in flies treated with both MA-CDs and CBZ. However, higher doses of CBZ and MA-CDs increased the climbing ability of the flies. In cognitive assays, CBZ at higher doses improved memory and learning in mutant flies, while MA-CDs also showed significant impact. MA-CDs were consumed at a higher rate than CBZ when incorporated into food. The green synthesized MA-CDs at its higher concentration has garnered its positive effect on the mutants along with the CBZ antiepileptic drug which also has shown its positive effects when different concentration of them were treated to the mutants.

## Introduction

Epilepsy is a chronic non-communicable neurological disorder characterized by a persistent predisposition to generate unprovoked seizures and independent of immediate central nervous system insults^1^. These seizures may result either from abnormal electrical activity in the brain, leading to involuntary movements, loss of consciousness, and cognitive impairments or genetic mutations, brain injury, infections, and developmental disorders^2^. The activities of seizures may range from staring spells to convulsions and are central to epilepsy, though they may also result from fever, injury, or metabolic disturbances^3^. Affecting ~ 50 million people worldwide, epilepsy constitutes a major public health burden, with an active prevalence of 0.64%, a lifetime prevalence of 0.76%, and an annual incidence of 61.4 per 100,000 individuals^4^. Its occurrence is notably higher among socioeconomically disadvantaged populations and increases with age which necessitates the requirement for deeper investigation^5^.

*Drosophila melanogaster* serves as a powerful genetic model, for studying human diseases^6^, including neurological disorders like epilepsy, due to its shared basic biology, physiology, and neurological properties. Notably, 75% of human disease-related genes are conserved in flies, of which, bang-senseless (*bss*) mutants exhibits seizure-like phenotypes triggered by mechanical stimuli such as vigorous shaking, leading to rapid muscle contractions followed by transient paralysis^7,8^. Para gene mutations, which encodes voltage-gated sodium channels, disrupts the balance of excitatory and inhibitory signaling in their nervous system^9^. Seizures also impact food intake, cognitive function, and motor abilities which in turn affect their motor control, with reduced climbing ability used as an indicator of motor dysfunction in epilepsy models^10–12^. *bss* mutants are also reported to show seizures resembling those of pharmacologically resistant epilepsies caused by mutation of the human Na_v_ SCN1A, the alpha subunit of the voltage-gated sodium channel Nav1.1^13^. The study of Drosophila bang-senseless mutants offers crucial insights into the genetic and molecular basis of epilepsy, providing a robust platform for research in seizure mechanisms and potential therapeutic approaches.

A prime example of such a therapy is carbamazepine (CBZ), a first-line medication used to manage partial seizures and primary generalized tonic–clonic seizures^14^. CBZ functions by inhibiting VGSC, stabilizing nerve cell membranes, and preventing the excessive neuronal firing responsible for seizures. While CBZ has proven effective and relevant in managing epilepsy, it has no therapeutic impact in 30–40% of patients with epilepsy^15^. This treatment gap reflects the need for alternative therapeutic strategies, including novel drug formulations and advanced delivery systems, to enhance efficacy and overcome drug resistance.

Nanotechnology has emerged as a promising field for novel epilepsy therapies^16^. Nanomaterials, including metal nanoparticles^17^, liposomes^18^, and polymeric nanoparticles^19^, have shown potential in drug delivery, neuroprotection, and targeted therapy for neurological disorders. Their tunable properties enable precise interaction with biological systems, improving therapeutic efficacy while minimizing side effects. Among these, carbon dots (CDs), due to their optical stability, biocompatibility, low toxicity, and ability to cross biological barriers, makes them ideal for biomedical applications^20^. Their synthesis follows two main approaches: top-down, which breaks down larger carbon structures but involves hazardous reagents, and bottom-up, which assembles smaller precursors and is more cost-effective and energy-efficient^21^. Among bottom-up methods, microwave-assisted synthesis is preferred for its speed and efficiency. Green synthesis, utilizing natural sources like plant extracts and biomass, offers an eco-friendly alternative, producing biocompatible CDs with minimal cytotoxicity^22–24^.

With increasing focus on sustainability, plant-derived CDs have gained attention due to their rich bioactive composition, enhancing their biomedical potential^25^. Various medicinal plants, including tulsi^26^, turmeric^27^, ginger^28^, neem^29^, and *Aloe barbadensis*^30^, have been explored for CD synthesis and their potential applications in nanomedicine, bioimaging, and drug delivery. Preliminary studies suggest CDs may exhibit neuro-protective^31^ and anti-epileptic properties by mitigating oxidative stress and interacting with neuronal pathways^32^. *Martynia annua*, or Cat’s claw, is an herbaceous plant of the *Martyniaceae* family, valued for its unique morphology and medicinal properties^33^. The leaves of *M. annua* are traditionally used for their anti-inflammatory, analgesic, antimicrobial, and anticonvulsant properties, aiding in pain relief, wound healing, and infection control^34^. It also supports digestion, detoxification, respiratory health, fertility, and is used as a remedy for snakebites and epilepsy^35^. In folk medicine, it is believed to help manage epilepsy and seizures by calming the nervous system^34^. Some studies also suggest that its bioactive compounds may have neuroprotective effects^36^. Hence, leaves of *M. annua* stands out as a potential precursor for the synthesis of CDs and to explore its possible biomedical applications.

This study presents the first report on the synthesis and characterization of MA-CDs derived from the leaf extract of *M. annua*. In addition to the synthesis process, the study investigates the effects of these MA-CDs and the drug CBZ on para^bss1^ epileptic mutants.

## Materials and methods

### Collection of plant material

The plant was collected from the region of Western Ghats in Karnataka, India. *Martynia annua*, with the accession number (IGGFWC/Marty-043) was identified and authenticated by Dr. Shivanand S. Bhat, taxonomist, Department of Botany, Smt. Indira Gandhi Government First Grade Women’s College, Sagar, Karnataka, India.

### Preparation of leaf extract

The freshly collected leaves were thoroughly washed with tap water, followed by distilled water. Subsequently, the leaves were dried in the shade and ground into a fine powder. 10 g of leaf powder was added to 100 ml of distilled water, to obtain the leaf extract. To facilitate the diffusion of plant compounds the mixture was kept in hot water bath for 1 h at 60 °C. The extract was allowed to cool and it was filtered through Whatman filter paper No.1, which was further stored at room temperature for future use^37^.

### Qualitative phytochemical analysis

The presence of phytochemicals in the aqueous leaf extract was assessed following a previously reported method^38^, as detailed in Table 1.

**Table 1:** Qualitative phytochemical analysis of aqueous extract.

Chemical constituent	Test	Observation	Result
Alkaloids	Wagner’s test: To the 2 ml of leaf extract, few drops of Wagner’s reagent were added	Formation of reddish-brown precipitate represents the presence of alkaloids	Absent
Flavonoids	Ferric chloride test: For the aqueous extract, few drops of FeCl_3_ (1%) was added	Appearance of blackish red color reveals the presence of flavonoids	Present
Glycosides	*Keller-Kiliani test:* To the 2 mL of extract, glacial acetic acid was added along with one drop ofFeCl_3_ (5%) and conc. H_2_SO_4_	The appearance of reddish-brown color at the junction of two liquid layers and bluish green color in the upper layer confirms the presence of glycosides	Absent
Phenols	*FeCl*_*3*_* test:* 5% of FeCl_3_ was added to the extract	The presence of blue-black color confirms the presence of phenols	Present
Saponins	*Frothing test:* The extract was diluted using distilled water (20 mL) and was shaken in a measuring cylinder for 15 min and was allowed to rest	Formation of foam around one cm confirms the presence of saponin	Present
Tannins	*Gelatin test:* Liquid gelatin was added to the extract	Appearance of white precipitate reveals the presence of tannins	Present
Terpenoids	*Salkowski tests:* 2 mL of conc. H_2_SO_4_ chloroform was added to the extract	The appearance of reddish-brown color confirms the presence of terpeniods	Present
Phytosterols	*Salkowski tests:*2 mL of chloroform and conc. H_2_SO_4_ was added to the extract	Red color formation for chloroform and the greenish yellow fluorescence in the acid layer indicates the presence of steroids	Present

### Quantitative phytochemical estimation

#### Estimation of total flavonoid content

The total flavonoid content (TFC) of the plant extract was determined using boric acid and oxalic acid, with quercetin as the standard. A stock solution of quercetin was prepared by dissolving 10 mg of quercetin in 10 mL of ethanol, and from this, serial dilutions were made to obtain concentrations of 20, 40, 60, 80, and 100 µg/mL. Similarly, 10 mg of the plant extract was dissolved in 10 mL of distilled water for sample preparation. For the assay, 1 mL of 0.1 M boric acid and oxalic acid was added to both the plant extract sample and the quercetin standard solutions. A blank was prepared using ethanol and the acids without the sample. The mixtures were incubated at room temperature for 15 min, and the absorbance was recorded at 410 nm using a UV–Vis spectrophotometer. A calibration curve was generated using the absorbance values of quercetin at different concentrations, which was subsequently used to determine the flavonoid content in the plant extract^39^.

#### Estimation of total phenolic content

The total phenolic content (TPC) of the leaf extract was determined using the Folin-Ciocalteu (FC) colorimetric method. For the extraction, 10 mg of plant powder was dissolved in 10 mL of distilled water and stirred for 1 h to ensure proper dissolution. Gallic acid was used as a standard for TPC quantification. A stock solution was prepared by dissolving 10 mg of gallic acid in 10 mL of ethanol, and from this, serial dilutions were made to obtain concentrations of 20, 40, 60, 80, and 100 µg/mL. For the assay, 2.5 mL of 10% (v/v) Folin-Ciocalteu reagent was added to the test tube and mixed thoroughly for 5 min. Following this, 0.5 mL of 0.5 M potassium iodide and 2 mL of 7.5% (w/v) sodium carbonate were added. The blank solution consisted of the same reagents without the sample. To determine the TPC of the extract, 0.5 mL of the sample was added to the reaction mixture, followed by 2.5 mL of 10% Folin-Ciocalteu reagent, mixed for 5 min, and then supplemented with 0.5 mL of 0.5 M potassium iodide and 2 mL of 7.5% sodium carbonate. The mixture was vortexed, incubated at room temperature for 30 min, and the absorbance was measured at 760 nm using a UV–Vis spectrophotometer (JASCO UV–VIS- NIR V-670). The experiment was conducted in triplicates, and a calibration curve was generated using the absorbance values of the gallic acid standards to determine the TPC of the extract^40^.

### Eco-Friendly preparation of MA-CDs via microwave method

The MA-CDs were synthesized using a microwave-assisted method^41^, with *M. annua* leaf extract as the precursor. A volume of 30 mL of the extract was subjected to microwave irradiation (800 W, IFB 20PM-MEC2) in a domestic microwave oven for 5 min, ensuring complete carbonization. Upon cooling down to room temperature, 10 mL of distilled water was added to the resulting MA-CDs, and the mixture was centrifuged at 10,000 rpm for 10 min. The supernatant was carefully collected and filtered through a 0.22 μm syringe filter to remove any residual impurities. The purified MA-CDs were subsequently stored at 4 °C and lyophilized as needed for further use.

### Physicochemical characterization of MA-CDs

Green-synthesized MA-CDs were characterized using a variety of analytical techniques. The optical properties of the MA-CDs were analyzed using UV–Visible spectroscopy with a JASCO UV–VIS NIR V-670 spectrophotometer (Hachioji, Tokyo, Japan). The measurements were conducted across a wavelength range of 200–800 nm, with a resolution of 1 nm^42^. Fourier Transform Infrared Spectroscopy (FT-IR) was conducted to identify the surface functional groups of the MA-CDs. The analysis was performed using a Nicolet iN10 instrument (Thermo Fisher Scientific, Waltham, Massachusetts, USA) over a spectral range of 400 to 4000 cm^−1^^43^. The particle size and surface morphology were analyzed via high-resolution transmission electron microscopy (HR-TEM) using a Jeol/JEM 2100 microscope (Tokyo, Japan). The zeta potential of the MA-CDs was measured using a Horiba SZ-100 instrument via dynamic light scattering (DLS) technology^44^. XRD analysis was carried out analyze the physical parameters of green synthesized MA-CDs. The analysis was performed with the scan range of 2θ = 10–80°, 30 mA, with a scan speed of 40 kV of 10°min^−1^ using Cu K-α as radiation source. Rigaku SmartLab SE X-ray Diffractometer, Rigaku Corporation in Tokyo, Japan. To examine the composition of MA-CDs, EDX analysis was performed using Thermo scientific, Noran (USA).

### Biological assays using* Drosophila*

#### Fly stock

The *Drosophila melanogaster* paralytic mutants (para^bss1^) involved in this study were obtained from the Department of Fly facility, National Centre for Biological Sciences (NCBS) in Bangalore, India. The mutant strains were cultured in our laboratory on standard wheat cream agar media containing yeast granules, with constant temperature of 22 ± 1 °C and relative humidity of 70–80%^45^.

#### Drug standardization

For the standardization of CBZ drug, Mohammad’s modified protocol was utilized ^46^. To find out the lethal concentration (LC_50_) of the drug the adult flies were exposed to different drug doses in the standard wheat cream agar media. Further, the dosage was finalized for 5 and 10 µg/mL, of media respectively. Similarly, the MA-CDs concentrations were standardized and the concentration was finalized to 5 and 10 µg/mL of media, respectively.

#### Treatment of drug CBZ and MA-CDs to the flies

The mutants were exposed 2 different concentrations of CBZ and MA-CDs such as 5 and 10 µg/mL, respectively. The 2 varied concentration drug and MA-CDs were added to the partially cooled media which reflects the final dosage concentration of fly food and was supplemented to the unmated male and virgin female flies, to study their effects.

#### Induction of seizure by vortex assay

A modified protocol of Moghimi & Harini, was used for the seizure induction in the control flies and the flies which were treated with different doses of CBZ and MA-CDs, via the vortex assay^47^. To perform the seizure induction via vortex assay, seven days old flies raised on standard and treated food were utilized. Group of ten flies were taken and anaesthetized using chloroform and after the recovery they were vortexed at a maximum speed for 60 s. Further, the flies were observed until they regained their posture and mobility.

#### Heat shock assay

**Scheme 1:**
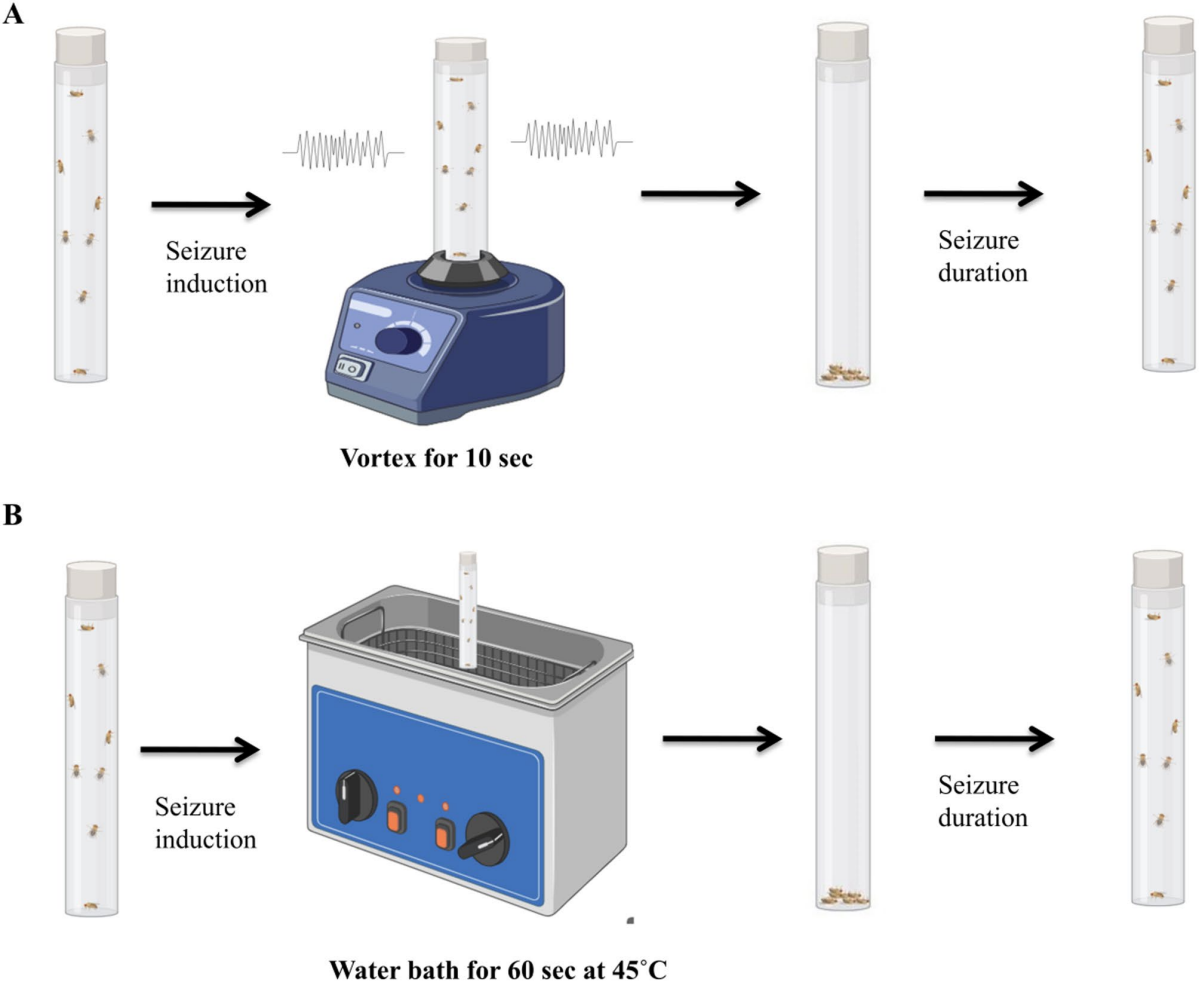
Seizure induction methods in para^bss1^ mutants (**A**) Represents the induction of seizures using vortex assay. The flies were vortexed for 10 s at maximum speed and the time required to recover was calculated. (**B**) Represents the induction of seizures using heat shock assay. The flies were kept in water bath at 45 °C for 60 s at maximum speed and the time required to recover was calculated.

A modified protocol of Mituzaite et al. was employed to optimize the assay conditions for this study^7^. To measure the seizure threshold and duration of recovery seven days old flies raised on standard and treated food, were kept in water bath at a constant temperature of 45 °C for 60 s. During this time, the flies were visually inspected every 10 s to monitor seizure activity. Seizures were identified by a loss of posture and random wing buzzing. After 60 s, the vials were removed from the water bath, and the duration of seizure activity was recorded during the recovery period, noting the time it took for the flies to regain normal posture and mobility (Scheme 1).

#### Climbing assay

The climbing assay was conducted following a method similar to the one described by Linderman et al.^48^. The locomotory activity of seven days old flies reared on standard and treated food was assessed by monitoring their vertical climbing using measuring cylinder. A group of thirty flies per condition was acclimated, gently tapped to the bottom of the glass cylinder, and then allowed to climb. The time taken by the flies to pass the 15 cm mark was recorded. Three replicates of thirty flies were tested for each condition.

#### Visual learning and memory assay

For the better understanding of neurological mechanisms underlying epilepsy and related cognitive impairments, visual learning and memory assays were conducted in mutant flies, following a modified method used earlier^49^. In this assay, control and treated flies were used for comparison. Two visual cues, blue and red light, were designated as visual cue A and cue B, respectively. An appetitive stimulus, represented by horizontal stripes, was also employed. Initially, the control and treated flies were exposed to the appetitive stimulus alongside cue A, and the same procedure was repeated for cue B, with each exposure lasting 1 min and conducted in triplicates. Subsequently, the flies were simultaneously exposed to both cue A and cue B to evaluate their memory of the visual cues. The preference index was calculated based on the movement of flies towards the respective visual cue using the following formula.

$${\text{Preference}} \;{\text{index}}\; = \;\frac{{{\text{Number}} \;{\text{of}} \;{\text{flies}}\;{\text{ in}}\;{\text{ cue}}\; {\text{A}}\; - \;{\text{Number}}\; {\text{of}}\;{\text{ flies}} \;{\text{in}}\; {\text{cue}}\; {\text{B}}}}{{{\text{Total}}\;{\text{ number}}\;{\text{ of}}\;{\text{ flies}}}}$$

#### Food intake assay

To evaluate the feeding behaviors of mutants the food intake assay was carried out using the modified protocol of Wong et al.^50^. Initially the seven days old flies were starvated for 30 min and later they were fed with the food prepared by mixing the orange-red dye. After, one hour of feeding the flies was homogenized using the phosphate buffer in homogenizer. Further, the homogenate was centrifuged for 13,000 rpm for 5 min to remove the cell debris and the homogenate was quantified spectrophotometrically by measuring absorbance at 508 nm.

#### Gut quantification

Gut quantification was carried out using the third instar larvae. The larvae were kept for starvation for half an hour and after the starvation the larvae were transferred to the media containing 0.5% of bromophenol blue salt and the different concentration of our drug and the MA-CDs, for 2 h. After, that the feeding was stopped and the larvae were taken out from the media and were homogenized using phosphate saline buffer and the absorbance was taken at 594 nm (Dare et al., 2021).

#### Cytotoxicity assay

Cytotoxicity of green synthesized MA-CDs were studied by detecting the amount of DNA damage happened in the human blood samples, with slight modifications^37^. Different concentrations (2.5, 5, 10 and 15 µg mL^−1^) of green synthesized MA-CDs were treated for the human blood cells for 24 h, in a CO_2_ incubator at 37 °C. To examine the toxic effects of MA-CDs DNA was isolated from human blood cells was extracted using phenol–chloroform method for both treated and the control samples, using 50 base pair DNA marker. Ethical clearance was obtained by Karnatak Institute for DNA Research (KIDNAR) under IEC Reference number KCTRI/EC-01-A/2024. Written consent was obtained from all individual participants included in the study. All procedures performed in studies involving human participants were in accordance with the Helsinki declaration as revised in 2013 and its later amendments.

### Statistical analysis

Data was analyzed using one way ANOVA for food intake and negative geotaxis assay and Two-way ANOVA was used for the vortex and heat shock assay using post hoc Tukey Test in Graph Pad Prism 8 with a significance level of *p* < 0.05. The biological assays were performed in triplicates and the results with error bars in graph are expressed with mean ± SD.

## Results and discussion

### Phytochemical analysis

#### Preliminary confirmation test

The aqueous leaf extract of *M. annua* primarily contains the phytochemicals such as flavonoids, terpenoids, phenols, tannins and saponin, respectively (Table 1). The aqueous leaf extract of *M. annua* contains phytochemicals similar to those isolated using different solvents such as methanol, ethanol and acetone^52^.

#### Total flavonoid estimation

**Fig. 1:**
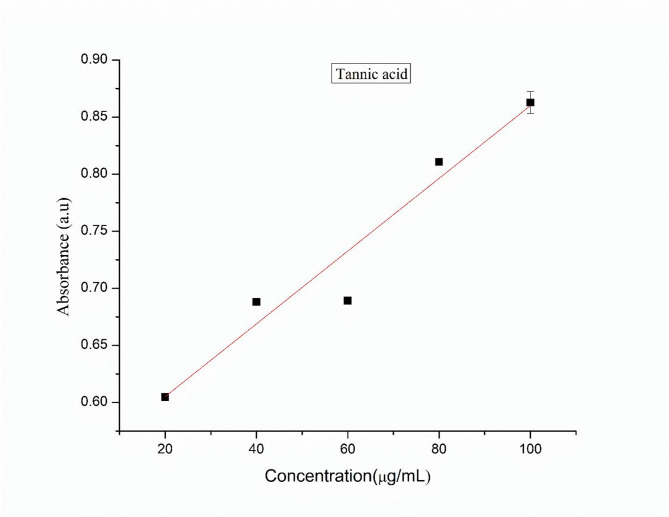
Calibration curve of the standard tannic acid.

Total flavonoid content present in the leaf extract was determined by using quercetin as a standard (Fig. 1). The total flavonoid content was calculated by using regression equation and expressed in terms of mg quercetin equivalents (QE) per gram of sample in dry weight (mg/g). The total amount of flavonoid present in the aqueous leaf extract was found to be 79.20 mg QE/g of extract.

#### Total phenolic content estimation

**Fig. 2:**
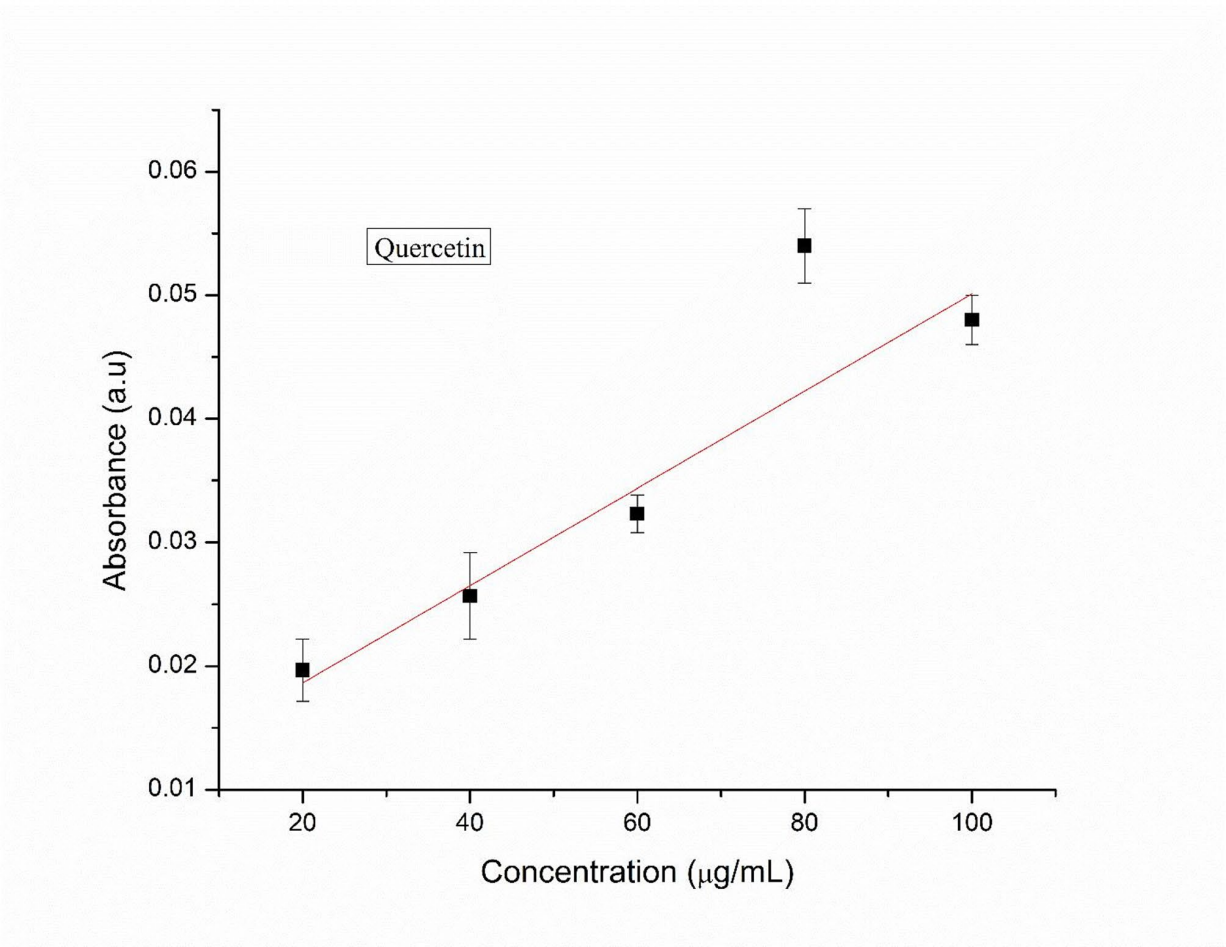
Calibration curve of the standard quercetin.

Total phenolic content was measured using the FC method using tannic acid as the standard (Fig. 2). Calibration curve was plotted by using the absorbance obtained from different concentrations of standard. The total phenolic content in the sample was calculated using the regression equation and expressed in terms of mg tannic acid equivalents (TAE) per gram of sample in dry weight (mg/g). The total phenolic content in the aqueous leaf extract was 65.78 mg tannic acid/g of extract.

The TFC and TPC values of *M. annua* leaf aqueous extract are comparable to other plant extracts, such as *Lagerstroemia speciosa* (269 mg TAE/g TPC and 214.32 mg QE/g TFC)^39^ and *Achyranthes aspera* (68.84 mg GAE/g TPC and 80 mg QE/g TFC), and *Achyranthes aspera* having 68.84 mg GAE/g and 80 QE/g respectively^53^.

### Synthesis of MA-CDs

**Scheme 2:**
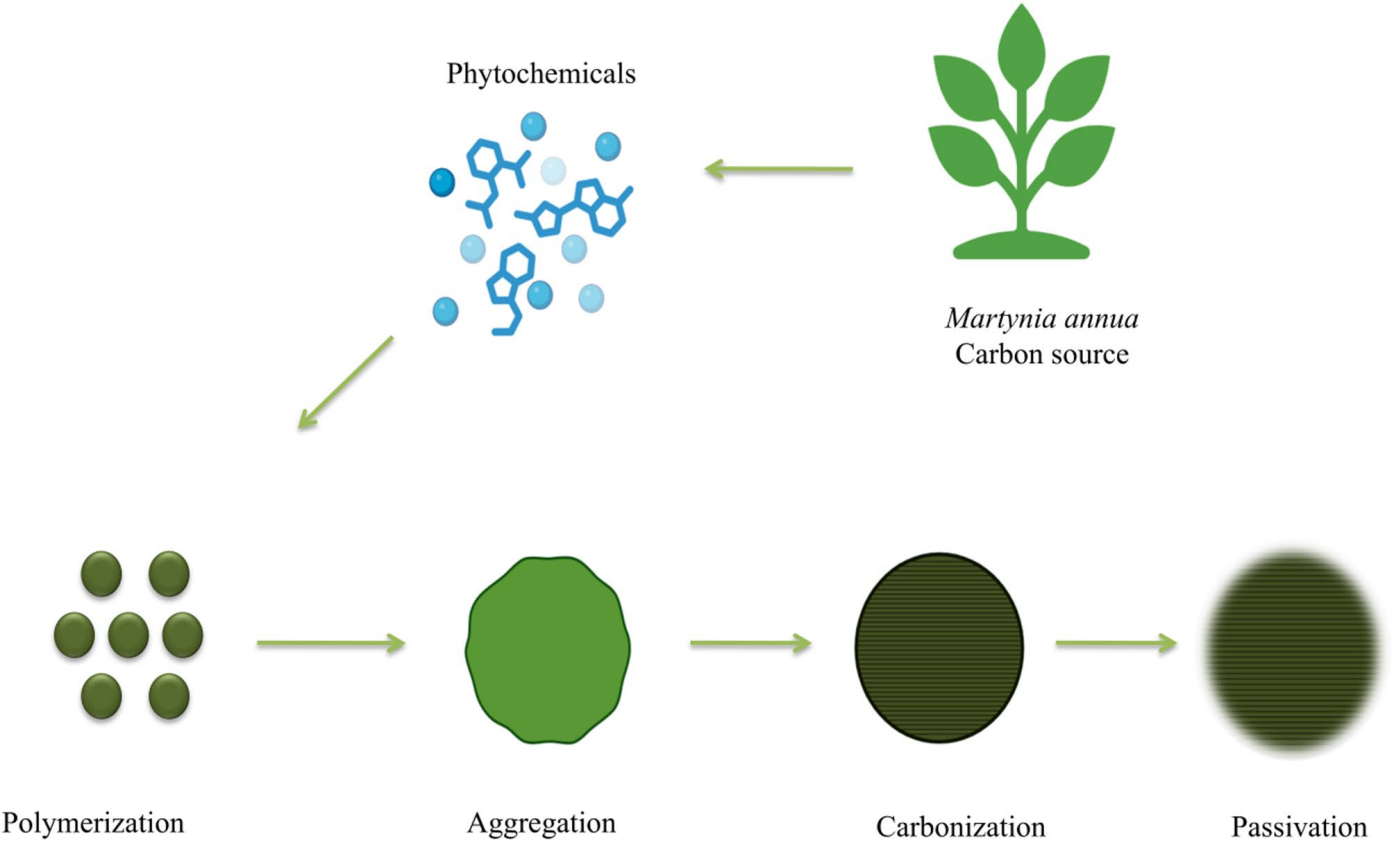
Schematic illustration of the possible mechanism involved in the synthesis of MA-CDs using *M. annua* as a carbon source.

In this study, the aqueous extract of *M. annua* leaves served as the sole carbon source without the addition of chemicals or passivation agents, ensuring a greener approach. Key phytochemicals, including chlorogenic acid, *p*-hydroxybenzoic acid, and sinapic acid, act as primary carbon sources in the formation of MA-CDs^54^. Fatty acids like palmitic and stearic acids contribute additional carbon, influencing size, shape, and stability^55^. Polymerization, aggregation, carbonization, and passivation were facilitated entirely by the natural components in the leaf extract^56^. Microwave heating enabled rapid and uniform energy distribution, converting biomolecules into MA-CDs (Scheme 2). During synthesis, carbonization formed nanostructures with unique optical and electronic properties^57^. Furthermore, functional groups like hydroxyl, carboxyl, and amine groups enhanced the water solubility, biocompatibility, and surface reactivity of MA-CDs^29^.

### Optical properties

**Fig. 3:**
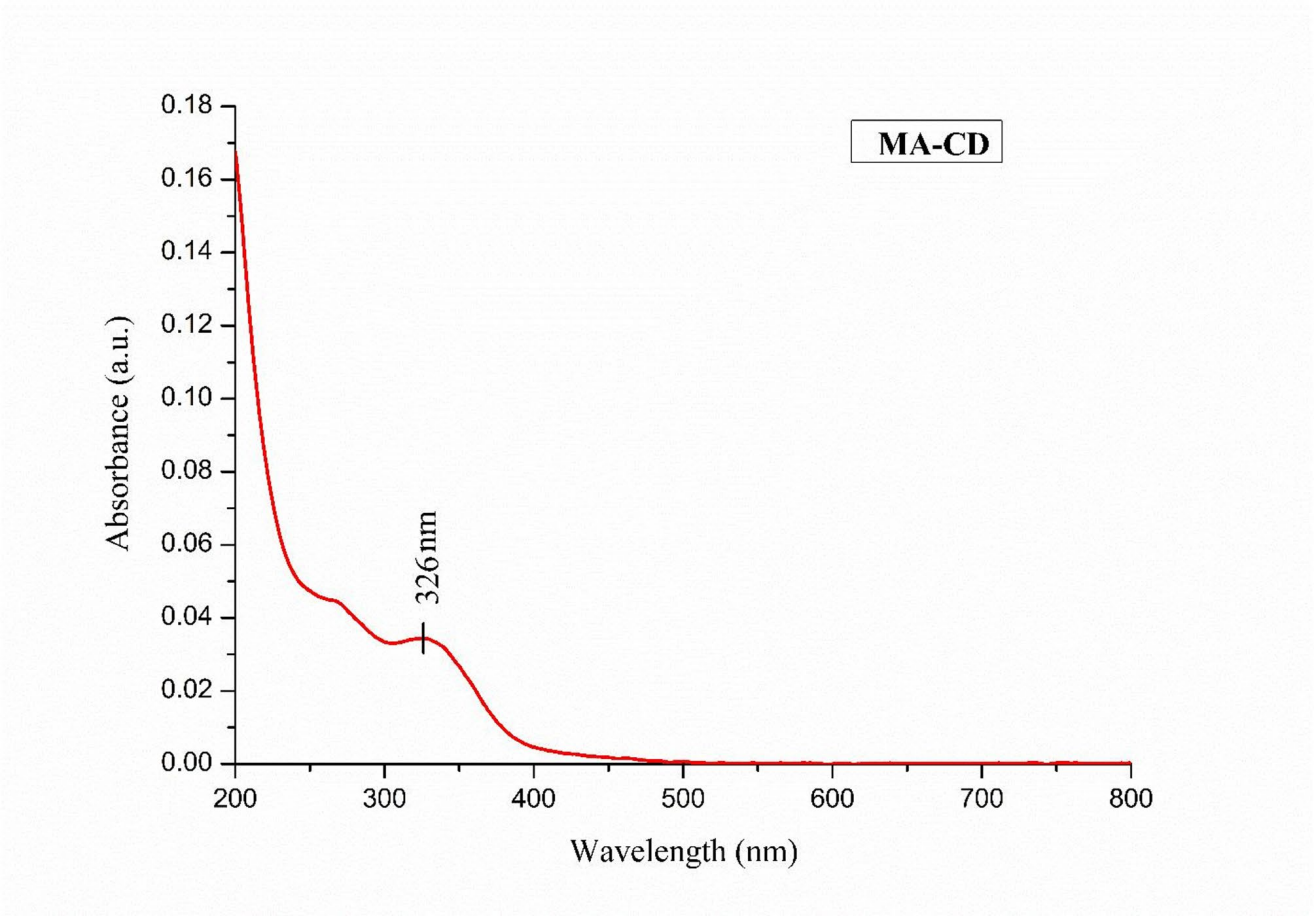
UV–Visible spectroscopic studies on the optical characteristics of MA-CDs.

UV–Visible spectroscopy was used to check the optical properties of MA-CDs which revealed the absorbance peak at 326 nm, which aligns to the transition n–π* associated with carboxyl groups with C = O, and another peak at 264 nm corresponding to π –π* of C = C on the surface of the MA-CDs. This distinctive peak signifies the functional groups that influence the optical properties of the prepared MA-CDs (Fig. 3). The findings were similar to CDs synthesized from *Centella asiatica* (213 nm and 322 nm)^41^.

### Microscopic, Spectroscopic and Surface characterization of MA-CDs

**Fig. 4:**
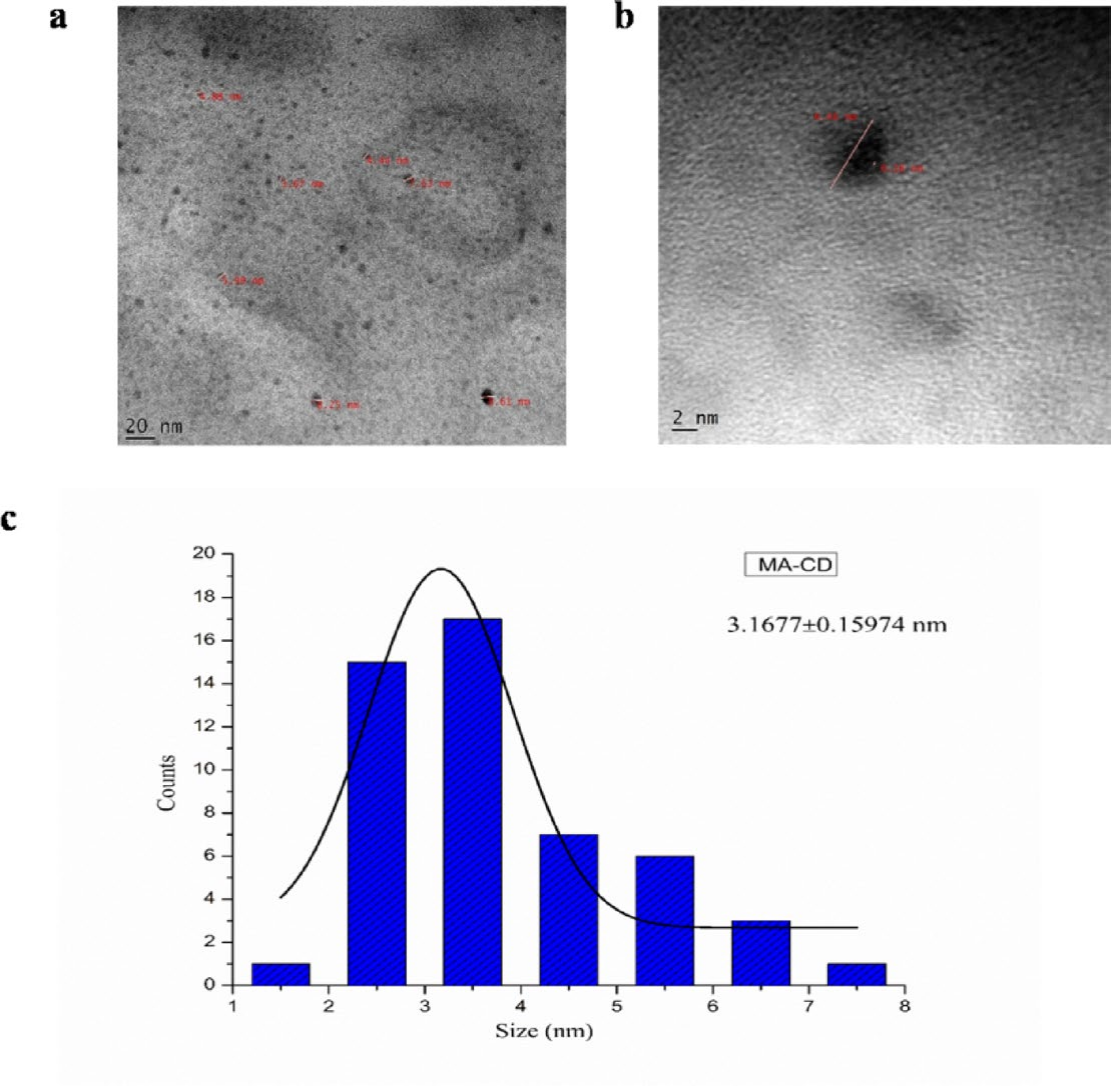
HR-TEM image showing the morphology of MA-CDs synthesized from the leaf extract of *M. annua* (**a**, **b**), (**c**) The histogram illustrates the particle size distribution of MA-CDs.

HR-TEM was utilized to visualize the morphological characteristics and size of the MA-CDs. The TEM images revealed that the particles are spherical (Fig. 4a and b), with an average diameter of 3.16 ± 0.15 nm (Fig. 4c) which is crucial for ensuring consistent optical and electronic properties across the MA-CDs. This is similar to CDs synthesized from *Azadirachta indica* via one-pot sand bath method (~ 3.5 nm)^29^.

**Fig. 5:**
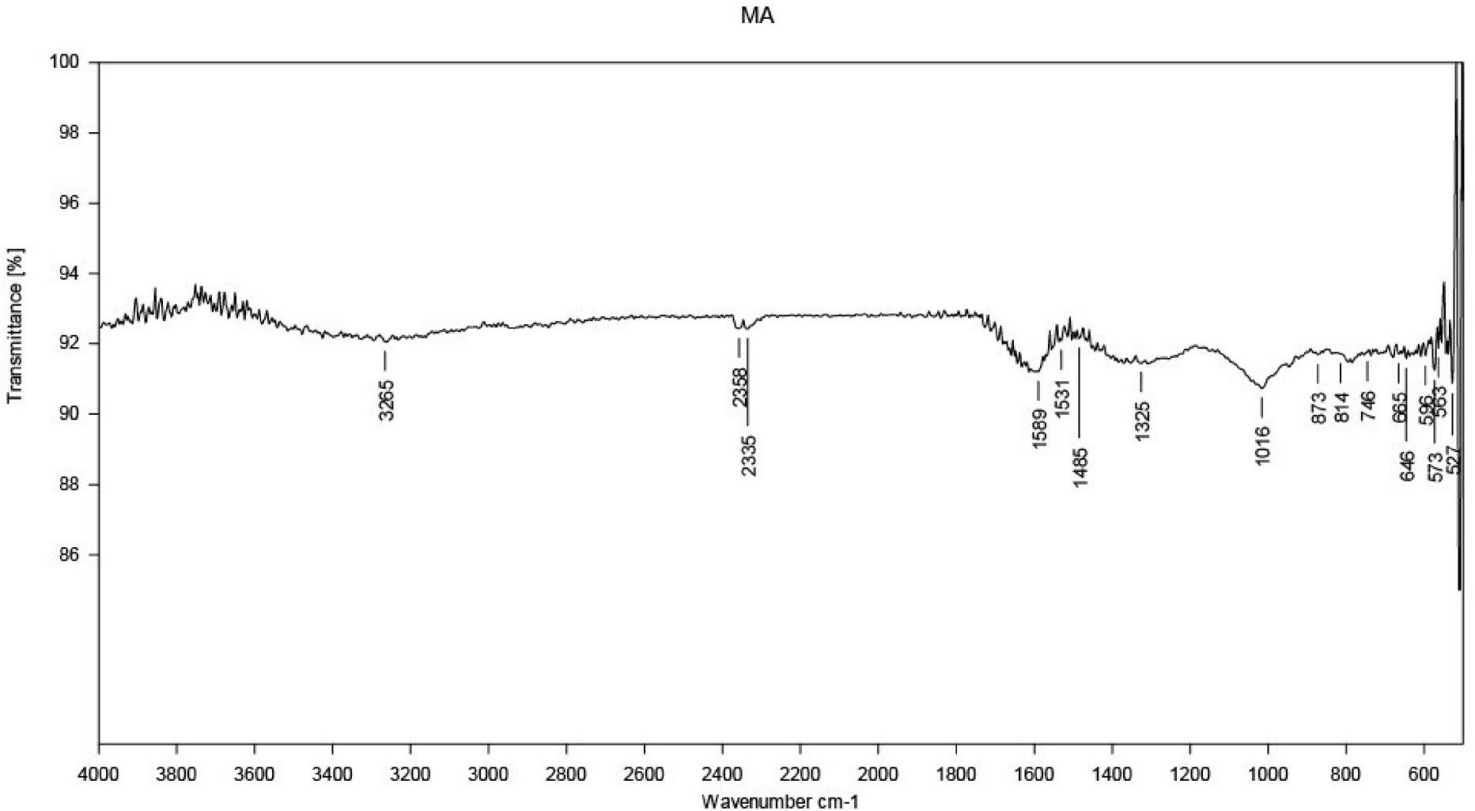
FTIR graph of MA-CDs fabricated from the leaf extract of *M. annua.*

FT-IR analysis is commonly used to identify the functional groups present on the surface of MA-CDs. The FT-IR spectrum of MA-CDs reveals several key absorption peaks. The peak at 3265 cm^−1^ aligns to the O–H stretching vibration, while the peak at 1589 cm^−1^ is associated with C = C stretching vibrations. Peaks at 1531 cm^−1^ and 1485 cm^−1^ represent C–H asymmetric stretching, while peaks at 1325 cm^−1^ and 1016 cm^−1^ correspond to C–O stretching vibrations, respectively (Fig. 5). Hence, there is high possibility of presence of phenolic, hydroxyl, carboxyl and carbonyl groups. This is consistent with CDs synthesized from *Citrus limetta* fruit extract ^58^.

**Fig. 6:**
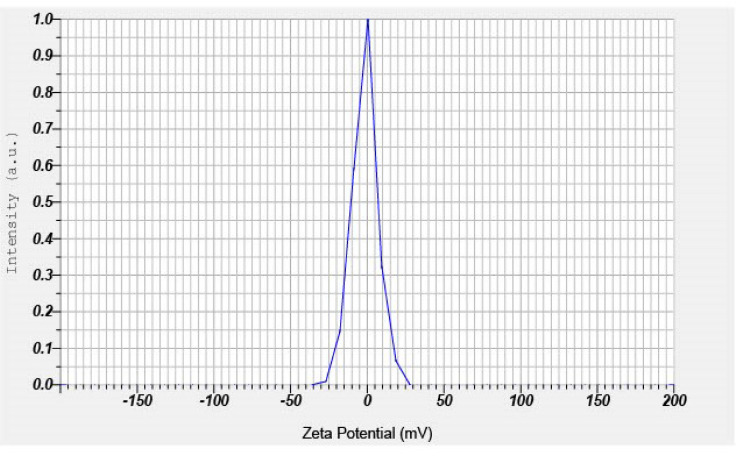
Zeta potential image of MA-CDs derived from the leaf extract of *M. annua.*

The zeta potential provides insight into the stability and surface charge of colloidal MA-CDs. Analysis of the synthesized MA-CDs showed a net negative charge of −1.6 mV (Fig. 6). Generally, particles with higher positive or negative surface charges exhibit stronger electrostatic repulsion, which enhances colloidal stability^59^. Hence, the low zeta potential of MA-CDs might induce agglomeration of particles over time. However, CDs with similar zeta potential (-2.1 mV) were found to possess multiple biomedical properties^57^. The stability of MA-CDs may be increased through surface functionalization with amine or carboxyl groups after synthesis or by using stabilizing additives during their formation^60^.

**Fig. 7:**
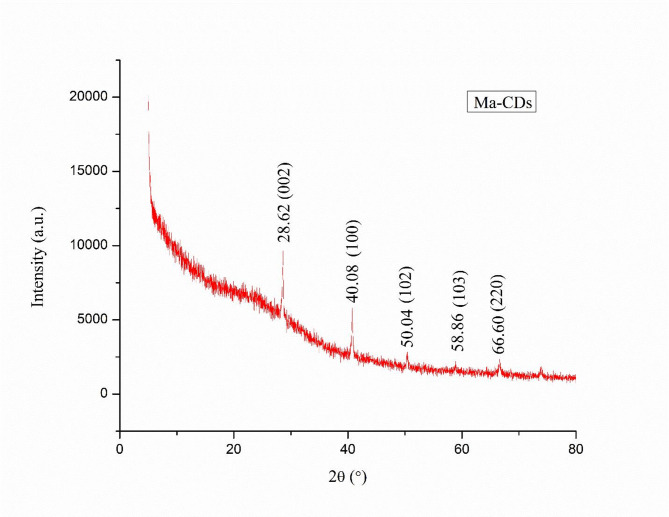
XRD spectrum of MA-CDs synthesized using the leaf extract of *M.annua.*

The XRD pattern (Fig. 7) depicts that the MA-CDs were polycrystalline in nature. The pattern shows a different diffraction peak at 2θ = 28.62°, 40.08°, 50.04°, 58.86° and 66.60° in which the first three planes corresponds to the sp^2^ graphitic carbon and the other two corresponds to the sp^3^ diamond carbon structure ^26^. Bragg’s equation was used to calculate the d- spacing values. The d-spacing values of MA-CDs at 2θ = 28.62°, 40.08°, 50.04°, 58.86° and 66.60° were found to be 3.11, 2.20, 1.80, 1.56, 1.40 Å, respectively. The calculated d-spacing values corresponds to the (002), (100), (102), (103) and (220) lattice planes representing the polycrystalline structure of MA-CDs. The results obtained in our study corresponds with the previous investigations, in which the CDs were synthesized using *Calotropis gigantean*^61^.

**Fig. 8:**
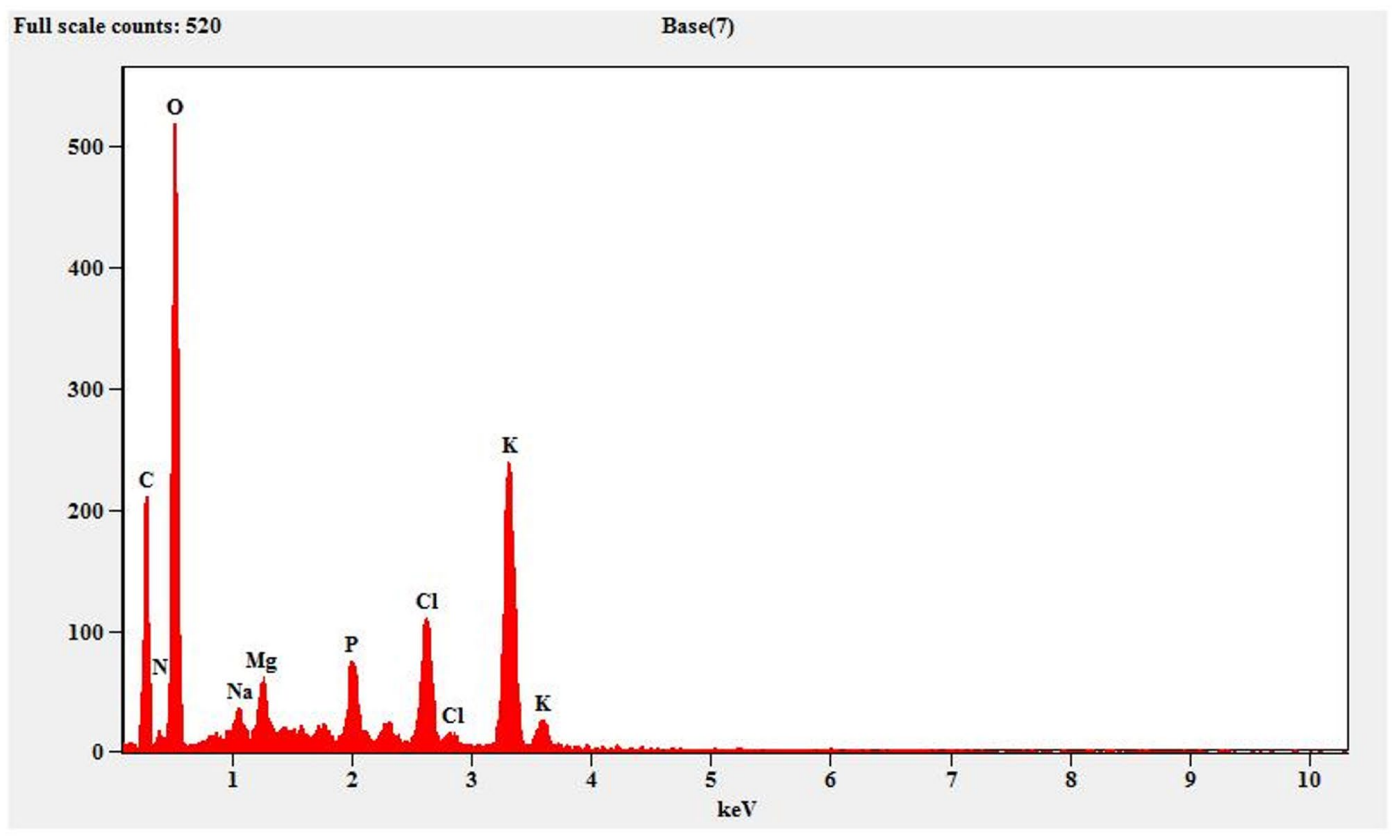
EDX spectrum of MA-CDs synthesized using the leaf extract of *M.annua.*

EDX analysis confirmed the presence of carbon and oxygen at 25.33 and 48.72% of weight percentage. Traces such as N, Na, Mg, P, Cl and K were also present which may have arisen which may be from the phytochemicals or impurities (Fig. 8). Similarly, the previous investigations have shown that the elements such as C and O were present at 50.08% and 28.89% along with traces such as N, Na, and Al^62^.

### Biological assays using* Drosophila* as a model

#### Induction of seizure via vortex assay and heat shock assay

Induction of seizures in para^bss1^ mutants can be effectively achieved through vortex assay and heat shock assay, which are commonly used to study seizure mechanisms and related neurological disorders. The adult vortex assay conducted on the para^bss1^ mutants revealed significant findings. The control group displayed a convulsion time of 95 s, a paralysis time of 99 s, and a recovery time of 97 s. Treatment with CBZ at 5 μg/mL notably reduced convulsion and paralysis times to 75 and 18 s, respectively, though the recovery time slightly increased to 85 s. At a higher dose of 10 μg/mL, CBZ further reduced convulsion and paralysis times to 63 and 16 s, respectively, while the recovery time decreased to 81 s.

In comparison, treatment with MA-CDs at 5 μg/mL reduced convulsion and paralysis times to 58 and 23 s, respectively, with a recovery time of 58 s. At 10 μg/mL, MA-CDs further decreased convulsion and paralysis times to 45 and 23 s, respectively, and shortened recovery time to 41 s.

**Fig. 9:**
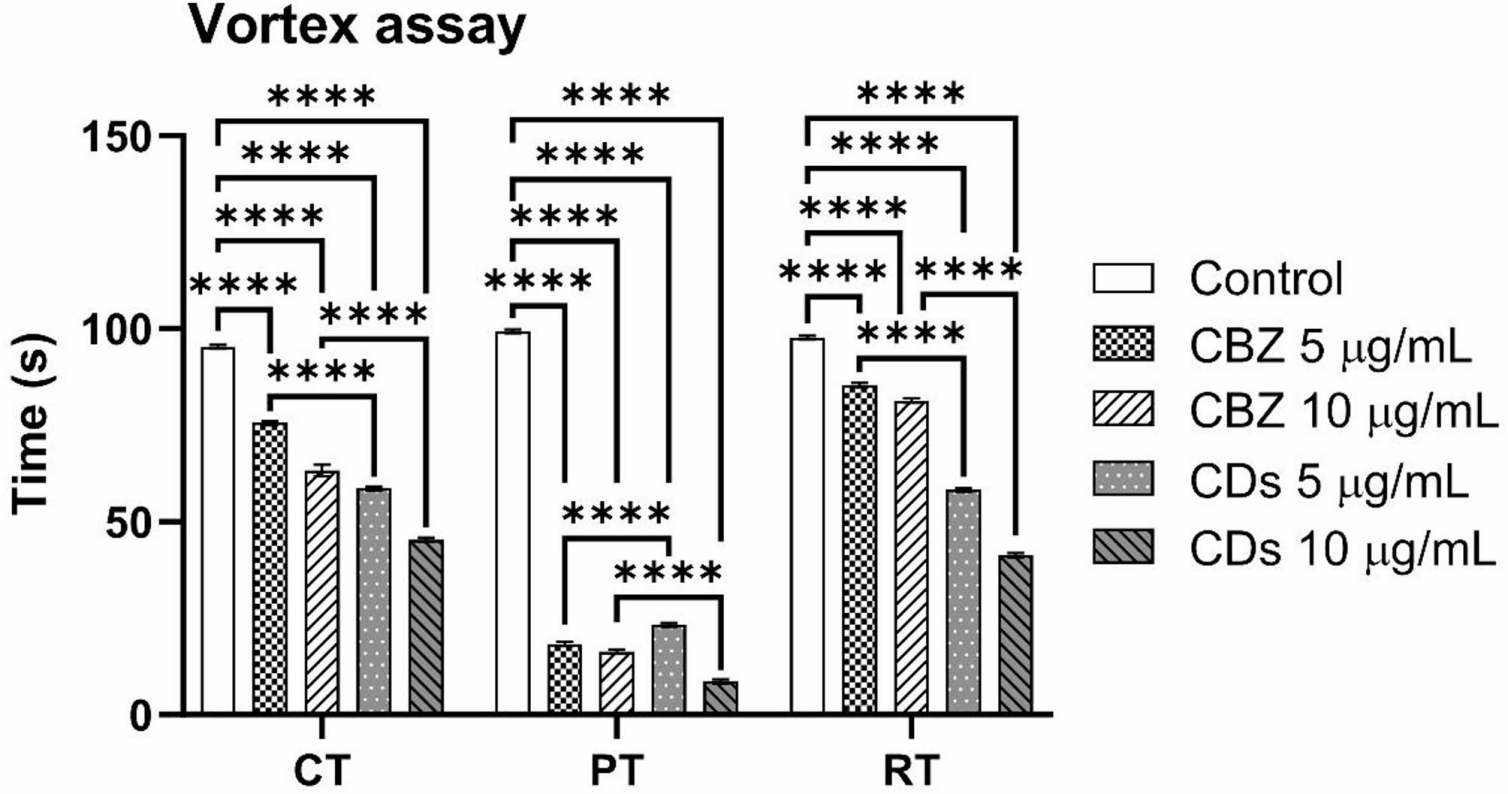
Adult vortex shock assay of para^bss1^ mutants treated with different concentrations of CBZ and MA-CDs. The bars represent the mean ± SD (****p < 0.0001, indicating a highly significant difference when compared with control and at similar concentrations).

These results, illustrated in Fig. 9, demonstrate that both CBZ and MA-CDs significantly influence the convulsion, paralysis, and recovery dynamics in para^bss1^ mutants after induced seizures. The dose-dependent effects highlight the therapeutic potential of CBZ and MA-CDs, while their varying efficacies underscore the complexity of their interactions at different concentrations.

**Fig. 10:**
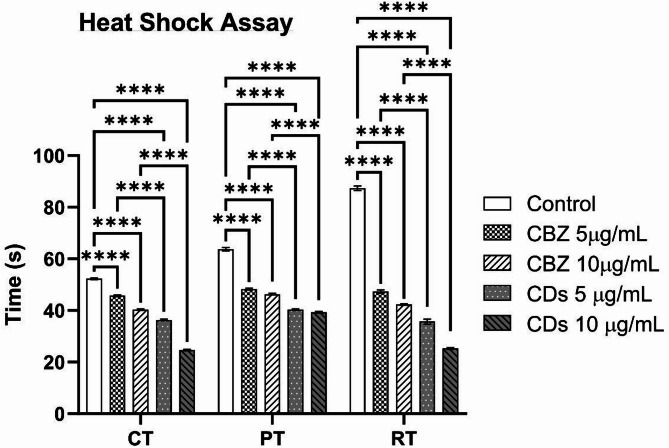
Adult heat shock assay of para^bss1^ mutants treated with different concentration of CBZ and MA-CDs. The bars represent the mean ± SD (****p < 0.0001 indicating a highly significant difference when compared with control and at similar concentrations).

The control flies subjected to the heat shock assay exhibited a convulsion time of 52 s, and a paralysis and recovery time of 63 and 87 s. Treatment with 5 and 10 μg/mL of CBZ and MA-CDs reduced the convulsion times to 45, 40, 36, and 24 s, respectively. Similarly, paralysis time decreased with increasing concentrations of both CBZ and MA-CDs compared to the control, with observed times of 63, 48, 46, 40, and 39 s. Furthermore, the recovery time following convulsion and paralysis also showed a dose-dependent reduction in treated groups relative to the control. The mean recovery times were recorded as 87, 47, 42, 35, and 25 s, respectively. These results demonstrate that both CBZ and MA-CDs effectively reduce seizure severity and recovery time in a concentration-dependent manner, as depicted in Fig. 10. Previous studies have demonstrated that para^bss^1 mutant flies treated with varying concentrations of sodium valproate and *Imperata cylindrica* extract exhibited significant, dose-dependent protection against chronic seizures^63^. Similarly, investigations on easily shocked (eas2) mutant flies have assessed the efficacy of multiple anticonvulsant drugs, including carbamazepine, ethosuximide, vigabatrin, gabapentin, and phenytoin. Among these, phenytoin exhibited the most significant effect in prolonging seizure recovery time. In contrast, carbamazepine, ethosuximide, and vigabatrin showed comparatively lower efficacy, whereas gabapentin was found to reduce the mean recovery time^64^.

#### Climbing assay

**Fig. 11:**
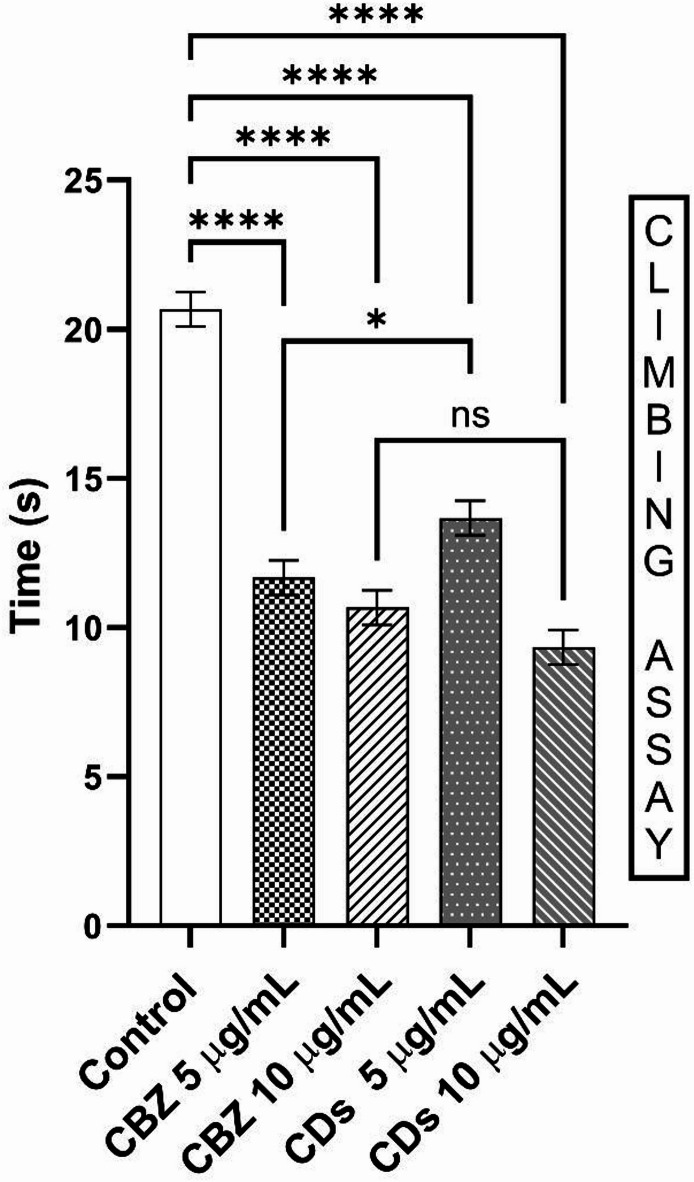
Climbing assay of par^bss1^ mutants treated with CBZ and MA-CDs. The bars represent the mean ± SD (****p < 0.0001, *p < 0.05, ^*ns*^*p* > 0.05, indicating a highly significant difference when compared with control).

Negative geotaxis, the natural tendency of *Drosophila* to climb against gravity, is a useful assay for assessing motor function and coordination^65^. In epilepsy models, such as para^bss1^, this climbing behavior is often impaired due to seizure-induced motor dysfunction^66^. The climbing assay conducted on para^bss1^ mutant assessed by measuring the time taken to traverse a distance of 15 cm (Fig. 11). In the control group, the flies took 20 s time to reach this distance of 15 cm. CBZ at a concentration of 5 μg/mL significantly improved climbing performance, reducing the time to mark the distance to 11 s. However, at a higher concentration of 10 μg/mL, the time to mark decreased slightly to 10 s, indicating an enhancement in climbing ability compared to the lower dose but still reflecting an improved performance relative to the control. Similarly, MA-CDs at 5 μg/mL also enhanced climbing performance, achieving the shortest time to mark of 13 s. Conversely, at 10 μg/mL, MA-CDs decreased the time to mark to 9 s, suggesting a lesser time taken in climbing ability at this higher concentration. Compared to previous studies, in which flies treated with silver nanoparticles synthesized using *Urtica diocia* exhibited improved climbing ability^67^. Compared to the normal *Drosophila* strain, flies treated with higher concentrations of Clobazam and Vigabatrin exhibited a reduced climbing ability relative to the control group. However, lower doses of these drugs did not produce any noticeable effects on locomotor performance^68^.

#### Visual cue-based learning and memory testing

Para^bss1^ flies show reduced visual learning abilities, as demonstrated in conditioned visual aversion assays, likely due to excessive neural firing disrupting synaptic plasticity. Both short-term and long-term memory retention are compromised, suggesting that seizure activity impairs the consolidation and recall of learned visual associations^69^. A differential impact of CBZ and MA-CDs on cognitive functions in mutant flies was assessed via visual cue-based learning and memory testing. In the control group, the preference index was 0.1, indicating baseline cognitive performance. Treatment with CBZ at 5 μg/mL significantly improved memory and learning, yielding a preference index of 0.2. However, at a higher concentration of 10 μg/mL CBZ resulted in a preference index of 0.3, reflecting a gain of cognitive preference. In contrast, MA-CDs at 5 μg/mL produced a preference index of 0.3. At 10 μg/mL MA-CDs yielded a preference index of 0.4 representing a significant enhancement in memory, comparable to that of the control group. These findings suggest that CBZ and MA-CDs enhances memory and learning abilities at both lower and higher concentrations. In the previous study by Shabir 2023 the normal drosophila flies treated with the zinc oxide nanoparticles have shown enhanced memory when exposed to light and dark conditions (Shabir et al. 2023).

#### Food intake assay

**Fig. 12:**
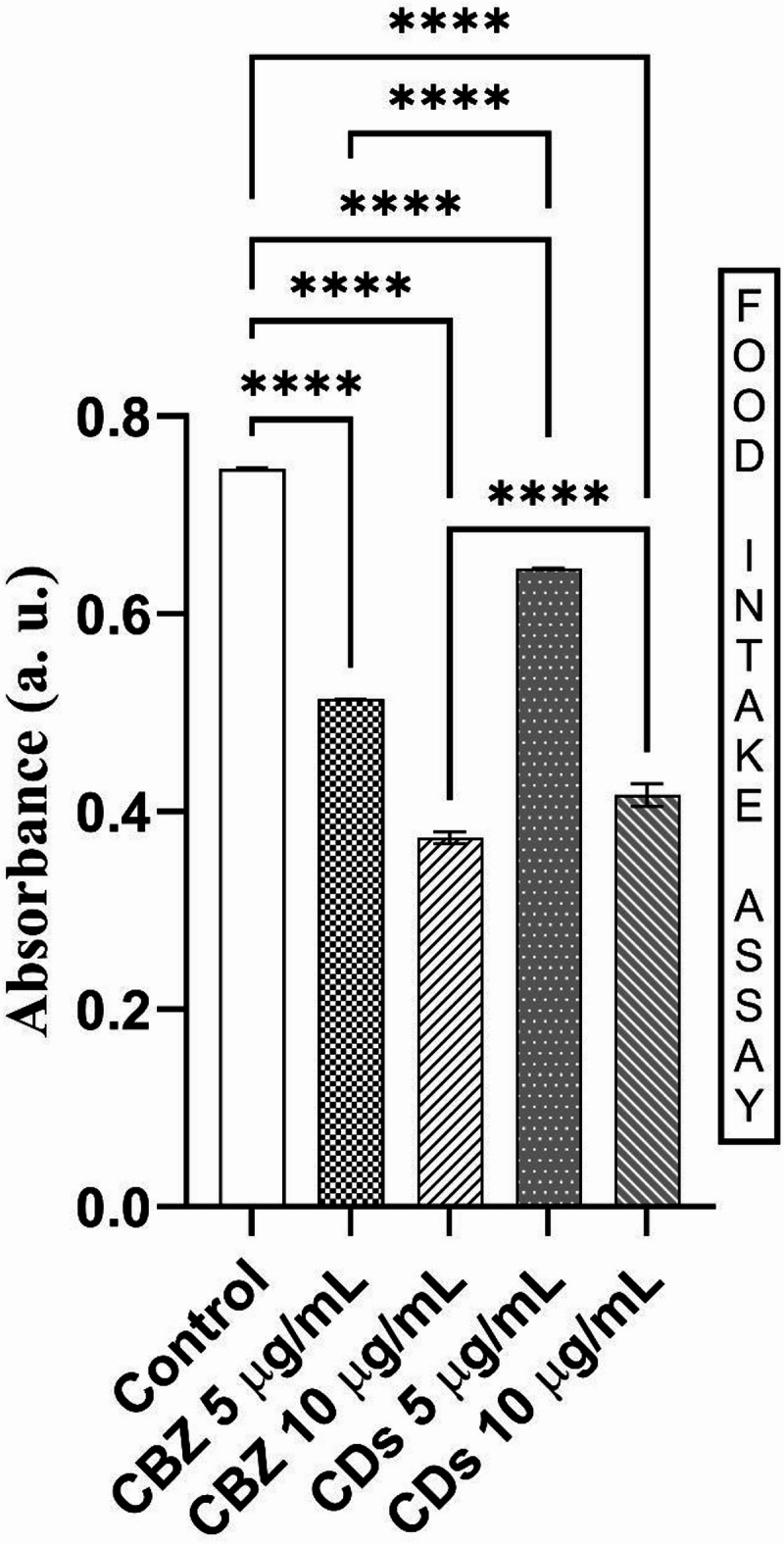
Food intake assay of para^bss1^ mutants treated with CBZ and MA-CDs. The bars represent the mean ± SD (****p < 0.0001, indicating a highly significant difference when compared at similar concentrations).

Food intake in the para^bss1^ was assessed using a colorimetric assay with orange-red dye, measuring absorbance at 508 nm to quantify consumption after exposure to various concentrations of CBZ and MA-CDs (Fig. 12). The control group, showed an absorbance of 0.75 nm, indicating substantial food intake due to the red dye. Flies treated with CBZ at 5 μg/mL displayed a significantly reduced absorbance of 0.51 nm, suggesting a marked suppression of feeding behavior. At a higher concentration of 10 μg/mL CBZ yielded an absorbance of 0.37 nm, indicating a dose-dependent decrease in food intake compared to the lower concentration. For the MA-CDs, treatment at 5 μg/mL, resulted in an absorbance of 0.64 nm, reflecting a bit reduction in food intake compared to the control, but this effect was less pronounced than that observed with CBZ. At 10 μg/mL, MA-CDs showed a slight increase in food intake with an absorbance of 0.42 nm, still lower than the control and 5 μg/mL, suggesting a mild inhibitory effect on feeding at elevated doses. Overall, these results indicate that CBZ strongly inhibits food intake in a dose-dependent manner, with higher concentrations allowing for slightly increased consumption. Conversely, MA-CDs increased food intake, but their inhibitory effect is less severe compared to that of CBZ. Both treatments significantly impact feeding behavior, with CBZ demonstrating a more substantial suppressive effect. Similarly, in case of previous studies, where the adult flies treated with silver nanoparticles have shown impairment in the food ingestion for the higher doses of silver nanoparticles (Raj et al. 2017).

#### Gut quantification assay

**Fig. 13:**
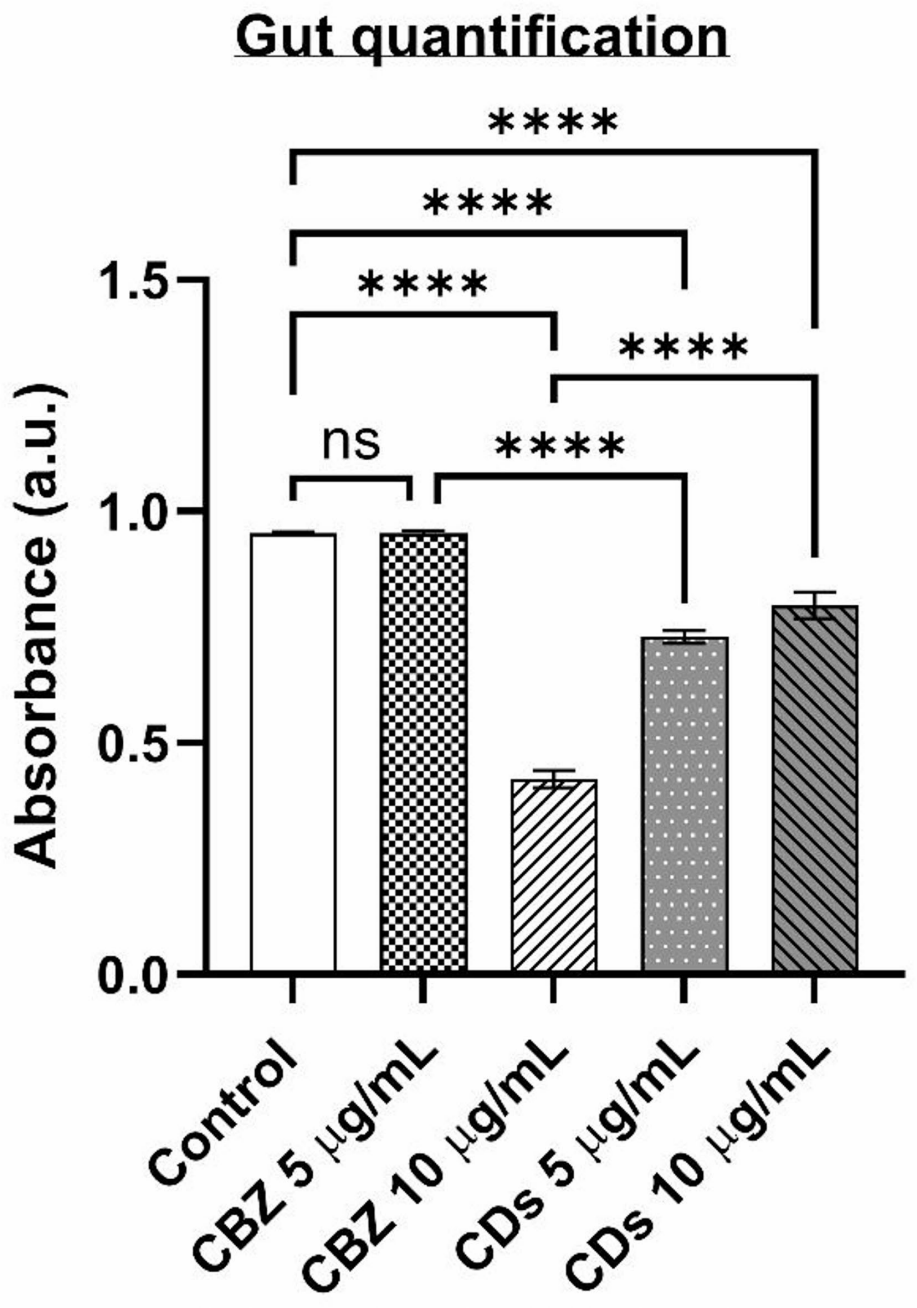
Gut quantification of para^bss1^ flies treated with different concentration of MA-CDs and CBZ The bars represent the mean ± SD (****p < 0.0001, ^*ns*^*p* > 0.05).

In the gut quantification assay, a slight variation in food intake was observed between control larvae and those treated with different concentrations of CDs and CBZ. The control group exhibited an absorbance of 0.95 nm (Fig. 13), which was comparable to that of larvae treated with 5 µg/mL of CDs. However, a reduction in food intake was noted in larvae exposed to 10 µg/mL of CDs. Similarly, the larvae treated with different concentrations of CBZ such as 5 µg/mL and 10 µg/mL treatment groups has recorded absorbance at 0.73 nm and 0.80 nm, respectively. Furthermore, larvae treated with higher concentrations of CDs demonstrated a more pronounced decrease in food intake compared to those exposed to lower concentrations of CDs and CBZ, indicating a dose-dependent effect on feeding behavior. Similarly, the previous investigations suggests that the flies treated with different concentrations of phenytoin and *A. senegalensis* leaf and bark stem extracts did not show any significant difference in the amount of food ingested^51^.

#### Cytotoxicity assay

**Fig. 14:**
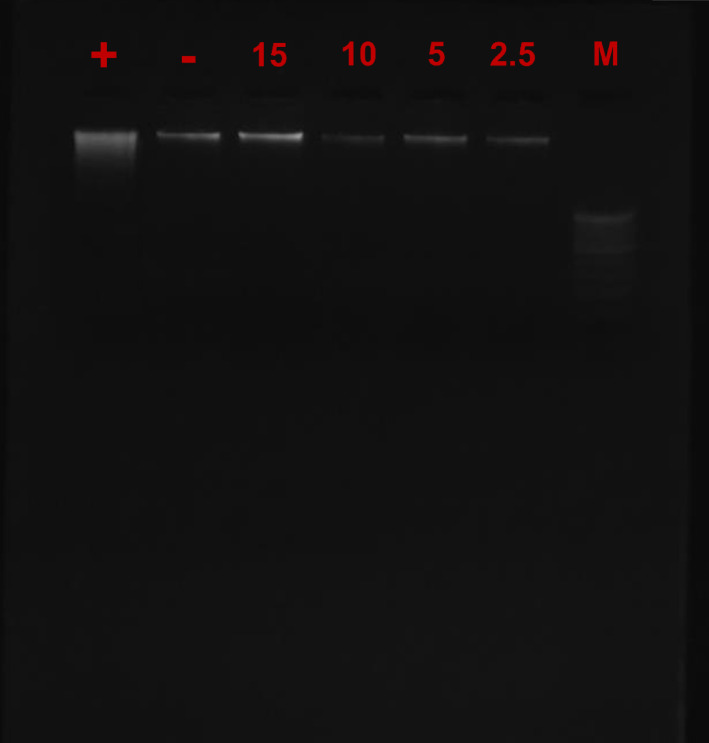
DNA fragmentation assay carried out using different concentrations of CDs to check the cytotoxicity. M, + , and—denotes ladder (100 bp), positive control and negative control respectively, while 2.5, 5, 10 and 15 refers to those treated with 2.5, 5, 10 and 15 µg/mL MA-CDs respectively.

The fragmentation in the DNA can happen because of the apoptosis or the toxic agents^70^. The cytotoxicity assay was performed to evaluate the toxicity of the green synthesized MA-CDs on the human blood cells. The dose dependent study of treatment of MA-CDs to the human blood cells have not shown any toxicity, which was indicated by the presence of intact bands in both treated and untreated cells (Fig. 14). The higher concentrations of the MA-CDs such as 15 µg/mL did not show any smear formation, indicating that the green synthesized MA-CDs did not exhibit any toxicity to the human blood cells. In contrast, the previous investigations suggests that the 30 μg/mL of CDs treated to the MCF-7 cells has caused the slight damage to DNA^71^.

The present study highlights the use of the para^bss1^ epileptic mutants, which exhibit abnormal brain phenotypes resulting from a mutation in the para gene of VGSC, located in neuronal cells^72^. The abnormal phenotype in the brain causes the mutation in the para gene. The neuro-pathological findings in this study align with prior research demonstrating bang-sensitive behavior and convulsions in *Drosophila melanogaster* seizure models with the para^bss1^ mutation, which over expresses the para gene. This mutation results in a reduced seizure-stimulation threshold, attributed to the increased expression of VGSCs in neuronal cells of the mutants, leading to heightened neural excitability and susceptibility to convulsive behavior^13^. Seizure behaviors in *Drosophila* originate in the brain’s epileptogenic zone and spread to secondary regions, such as motor centers and cognitive areas like the mushroom bodies^73^. This spread disrupts motor function and impairs the recognition and recall, ultimately causing cognitive deficits^74^.

**Scheme 3:**
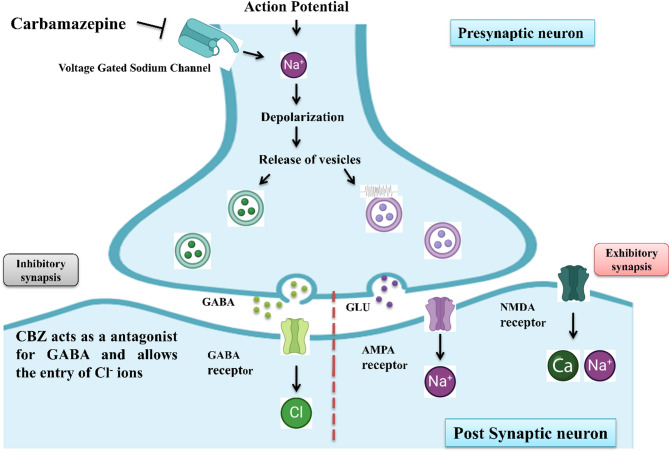
Mechanism of action of CBZ.

Both treatments CBZ and MA-CDs exhibited dose-dependent reductions in convulsion and paralysis times while promoting faster recovery compared to controls. MA-CDs showed comparable or superior efficacy in reducing convulsion times and enhancing recovery at higher concentrations. The climbing assay further confirmed improved motor performance with both treatments, with CBZ and MA-CDs enhancing climbing ability at increasing doses. Visual cue-based learning and memory testing indicated cognitive improvements in treated flies, with MA-CDs displaying a greater enhancement in memory at higher doses. In the food intake assay, CBZ significantly suppressed feeding behavior, while MA-CDs exhibited a milder effect. The gut quantification assay also reflected dose-dependent reductions in food intake with both treatments. These findings suggest that MA-CDs possess promising therapeutic potential for seizure management with minimal cytotoxic effects. CBZ is an anticonvulsant which modulates neurotransmitter release and may reduce appetite by affecting the central nervous system^75^, while MA-CDs might alter feeding behavior through their impact on cellular metabolism or stress responses. Both drugs likely suppress feeding due to their sedative or metabolic effects. The mechanism of action of CBZ is shown in the Scheme 3. Primarily CBZ binds to the inactivated VGSC, which in turn results the slows down of neuronal activity. Along with that, it also reduces the flow of Ca^+2^ and Na^+^ across the neural membrane. CBZ acts as a antagonist for GABA and allows the entry of Cl- ions^76^.

## Conclusion

The study successfully demonstrated the eco-friendly synthesis of MA-CDs using aqueous extract, revealing significant insights into their structural and functional properties. The MA-CDs showed distinct optical characteristics, nanoscale size, and moderate stability. Their administration in the para^bss1^, along with the antiepileptic drug CBZ, provided valuable information on seizure management. While CBZ enhanced learning and memory in the mutant flies, the MA-CDs primarily demonstrated an antioxidant effect, reducing oxidative stress markers. This suggests that green-synthesized MA-CDs hold promise as a complementary option in managing epilepsy, although further research is essential to optimize their efficacy and fully integrate them into therapeutic protocols for seizure and oxidative stress reduction. The plant *Martynia annua* is known for its antiepileptic properties, attributed to the phytochemicals present in its leaves. These bioactive compounds formed the basis for utilizing the aqueous leaf extract of *M. annua* in the synthesis of CDs. Consequently, the CDs synthesized from *M. annua* leaf extract are hypothesized to exhibit enhanced efficacy in the treatment of epilepsy compared to other conventionally synthesized CDs, owing to the inherent therapeutic potential of the plant’s phytoconstituents.

## Data Availability

All data supporting the findings of this study are available within this article. All other relevant data will be available from the corresponding author upon reasonable request.

## Abbreviations

MA-CDs: Carbon dots
CBZ: Carbamazepine
para^bss1^: Para bang senseless
FTIR: Fourier transform infrared spectroscopy
HR-TEM: High resolution transmission electron microscopy
CT: Convulsion time
PT: Paralysis time
RT: Recovery time
VGSC: Voltage gated sodium channel

## References

[1] Fisher, R. S. et al. Epileptic seizures and epilepsy: Definitions proposed by the international league against epilepsy (ILAE) and the international bureau for epilepsy (IBE). *Epilepsia* **46**, 470–472 (2005). 15816939 10.1111/j.0013-9580.2005.66104.x

[2] Reynolds, E. H. The ILAE/IBE/WHO global campaign against epilepsy: Bringing epilepsy “out of the shadows”. *Epilepsy Behav.* **1**, S3–S8 (2000). 12609455 10.1006/ebeh.2000.0104

[3] Hoxhaj, P. et al. Investigating the impact of epilepsy on cognitive function: A narrative review. *Cureus*10.7759/cureus.41223 (2023). 37525802 10.7759/cureus.41223 PMC10387362

[4] Fiest, K. M. et al. Prevalence and incidence of epilepsy: A systematic review and meta-analysis of international studies. *Neurology* **88**, 296–303 (2017). 27986877 10.1212/WNL.0000000000003509 PMC5272794

[5] Beghi, E. & Giussani, G. Aging and the epidemiology of epilepsy. *Neuroepidemiology* **51**, 216–223 (2018). 30253417 10.1159/000493484

[6] Shettar, A. K., Vedamurthy, B. & Yarajarla, R. B. Impact of dietary restriction on lifespan in different species of *Drosophila*. *Int. J. Pharm. Biol.* **9**, 1280–1290 (2019).

[7] Mituzaite, J., Petersen, R., Claridge-Chang, A. & Baines, R. A. Characterization of seizure induction methods in *Drosophila*. *eNeuro*10.1523/ENEURO.0079-21.2021 (2021). 34330816 10.1523/ENEURO.0079-21.2021 PMC8387149

[8] Pandey, U. B. & Nichols, C. D. Human disease models in *Drosophila melanogaster* and the role of the fly in therapeutic drug discovery. *Pharmacol. Rev.* **63**, 411–436 (2011). 21415126 10.1124/pr.110.003293 PMC3082451

[9] Ravenscroft, T. A. et al. *Drosophila* voltage-gated sodium channels are only expressed in active neurons and are localized to distal axonal initial segment-like domains. *J. Neurosci.* **40**, 7999–8024 (2020). 32928889 10.1523/JNEUROSCI.0142-20.2020 PMC7574647

[10] Madabattula, S. T. et al. Quantitative analysis of climbing defects in a drosophila model of neurodegenerative disorders. *JOVE*10.3791/52741-v (2015). 26132637 10.3791/52741 PMC4544889

[11] Kasuya, J., Johnson, W., Chen, H.-L. & Kitamoto, T. Dietary supplementation with milk lipids leads to suppression of developmental and behavioral phenotypes of hyperexcitable *Drosophila mutants*. *Neuroscience* **520**, 1–17 (2023). 37004908 10.1016/j.neuroscience.2023.03.027 PMC10200772

[12] Novak, A., Vizjak, K. & Rakusa, M. Cognitive impairment in people with epilepsy. *JCM* **11**, 267 (2022). 35012007 10.3390/jcm11010267 PMC8746065

[13] Parker, L., Padilla, M., Du, Y., Dong, K. & Tanouye, M. A. *Drosophila* as a model for epilepsy: BSS is a gain-of-function mutation in the para sodium channel gene that leads to seizures. *Genetics* **187**, 523–534 (2011). 21115970 10.1534/genetics.110.123299 PMC3030494

[14] Hashim, I. A. Therapeutic drugs and toxicology testing. In *Tutorials in clinical chemistry* 375–418 (Elsevier, Amsterdam, 2024). 10.1016/B978-0-12-822949-1.00020-6.

[15] Duggan, M. Epilepsy and its effects on children and families in rural Uganda. *Afr. H. Sci.* **13**, 613–623 (2013). 10.4314/ahs.v13i3.14 PMC3824457 24250298

[16] Bonilla, L. et al. Biodegradable nanoparticles for the treatment of epilepsy: From current advances to future challenges. *Epilepsia Open* **7**, 121 (2022). 10.1002/epi4.12567 PMC9340299 34862851

[17] Singh, S. R. et al. The effect of *Clitoria ternatea* L. flowers-derived silver nanoparticles on A549 and L-132 human cell lines and their antibacterial efficacy in *Caenorhabditis elegans* in vivo. *Hybrid Adv.* **8**, 100359 (2025).

[18] Danaei, M. et al. Impact of particle size and polydispersity index on the clinical applications of lipidic nanocarrier systems. *Pharmaceutics* **10**, 57 (2018). 29783687 10.3390/pharmaceutics10020057 PMC6027495

[19] Musumeci, T., Bonaccorso, A. & Puglisi, G. Epilepsy disease and nose-to-brain delivery of polymeric nanoparticles: An overview. *Pharmaceutics* **11**, 118 (2019). 30871237 10.3390/pharmaceutics11030118 PMC6471219

[20] Belkahla, H. et al. Carbon dots, a powerful non-toxic support for bioimaging by fluorescence nanoscopy and eradication of bacteria by photothermia. *Nanoscale Adv.* **1**, 2571–2579 (2019). 36132715 10.1039/c9na00140a PMC9418816

[21] Li, H. et al. Water-soluble fluorescent carbon quantum dots and photocatalyst design. *Angew. Chem. Int. Ed.* **49**, 4430–4434 (2010). 10.1002/anie.200906154 20461744

[22] Devi, P., Saini, S. & Kim, K.-H. The advanced role of carbon quantum dots in nanomedical applications. *Biosens. Bioelectron.* **141**, 111158 (2019). 31323605 10.1016/j.bios.2019.02.059

[23] Gedda, G., Lee, C.-Y., Lin, Y.-C. & Wu, H. Green synthesis of carbon dots from prawn shells for highly selective and sensitive detection of copper ions. *Sens. Actuators B Chem.* **224**, 396–403 (2016).

[24] Zulfajri, M. et al. Plant part-derived carbon dots for biosensing. *Biosensors* **10**, 68 (2020). 32560540 10.3390/bios10060068 PMC7345696

[25] Thokchom, B., Bhavi, S. M., Abbigeri, M. B., Shettar, A. K. & Yarajarla, R. B. Green synthesis, characterization and biomedical applications of *Centella asiatica*-derived carbon dots. *Carbon Lett.*10.1007/s42823-023-00505-3 (2023).

[26] Doshi, K. & Mungray, A. A. Bio-route synthesis of carbon quantum dots from Tulsi leaves and its application as a draw solution in forward osmosis. *J. Environ. Chem. Eng.* **8**, 104174 (2020).

[27] Yan, H. et al. Green synthesis of carbon quantum dots from plant turmeric holds promise as novel photosensitizer for in vitro photodynamic antimicrobial activity. *J. Mater. Res.* **22**, 17–34 (2023).

[28] Li, J. et al. Green preparation of ginger-derived carbon dots accelerates wound healing. *Carbon* **208**, 208–215 (2023).

[29] Gedda, G. et al. Green synthesis of multi-functional carbon dots from medicinal plant leaves for antimicrobial, antioxidant, and bioimaging applications. *Sci. Rep.* **13**, 6371 (2023). 37076562 10.1038/s41598-023-33652-8 PMC10115846

[30] Malavika, J. P. et al. A sustainable green synthesis of functionalized biocompatible carbon quantum dots from Aloe barbadensis Miller and its multifunctional applications. *Environ. Res.* **200**, 111414 (2021). 34052245 10.1016/j.envres.2021.111414

[31] Zhang, Y. et al. The neuroprotective effect of pretreatment with carbon dots from Crinis Carbonisatus (carbonized human hair) against cerebral ischemia reperfusion injury. *J. Nanobiotechnol.* **19**, 257 (2021). 10.1186/s12951-021-00908-2 PMC8399708 34454522

[32] Azab, M. M., Rizk, M., Abdel Ghany, N. A., Mohamed, O. M. & Shaaban Farag, A. Zirconia nanoparticles/carbon quantum dots modified carbon paste electrode as electrochemical sensor for determination of antiepileptic drug eslicarbazepine acetate in bulk and pharmaceutical dosage form. *Measurement* **238**, 115400 (2024).

[33] Gupta, P. P. et al. Therapeutic potential of *Martynia annua*. *EJPMR* **8**, 304 (2021).

[34] Rameshroo, K., Prasad, P., Satapathy, T. & Roy, A. *Martynia annua*: An overview. *PBJ* **1**, 07–10. 10.20510/ukjpb/1/i1/91102 (2013).

[35] Gupta, R. K. & Deogade, M. A critical review on ethnobotanical, phytochemical and pharmacological investigations of *Martynia annua* Linn. *IJAM* **9**, 136–143 (2018).

[36] Del Río, C. & Segura-Carretero, A. Neuroprotection with bioactive compounds. *Nutrients* **15**, 4612 (2023). 37960265 10.3390/nu15214612 PMC10647415

[37] Abbigeri, M. B. et al. Potential in vitro antibacterial and anticancer properties of biosynthesized multifunctional silver nanoparticles using *Martynia annua* L. leaf extract. *Nano-Struct. Nano-Objects* **39**, 101320 (2024).

[38] Pujar, R., Pujari, N., Shettar, A. K., Hoskeri, J. H. & Ramesh, B. Y. GC-MS based phytochemical profiling and investigation of in vitro pharmacological activity of *Croton sparsiflorus*. *Res. J. Biotech.* **17**, 8–18 (2022).

[39] Singh, S. R. et al. A study on the antioxidant, cytotoxicity, and coagulation potential of carbon quantum dots derived from the leaves of *Lagerstroemia speciosa*. *Hybrid Adv.* **10**, 100430 (2025).

[40] Siddiqui, N., Rauf, A., Latif, A. & Mahmood, Z. Spectrophotometric determination of the total phenolic content, spectral and fluorescence study of the herbal Unani drug Gul-e-Zoofa (*Nepeta bracteata* Benth). *J. Taibah Univ. Med. Sci.* **12**, 360–363 (2017). 31435264 10.1016/j.jtumed.2016.11.006 PMC6694887

[41] Thokchom, B., Bhavi, S. M., Abbigeri, M. B., Shettar, A. K. & Yarajarla, R. B. Green synthesis, characterization and biomedical applications of *Centella asiatic*a-derived carbon dots. *Carbon Lett.* **33**, 1057–1071 (2023).

[42] Bhavi, S. M. et al. Green synthesis, characterization, antidiabetic, antioxidant and antibacterial applications of silver nanoparticles from *Syzygium caryophyllatum* (L.) Alston leaves. *Process Biochem.* **145**, 89–103 (2024).

[43] Bhavi, S. M. et al. Biogenic silver nanoparticles from *Simarouba glauca* DC leaf extract: Synthesis, characterization, and anticancer efficacy in lung cancer cells with protective effects in *Caenorhabditis elegans*. *Nano TransMed* **3**, 100052. 10.1016/j.ntm.2024.100052 (2024).

[44] Bhavi, S. M. et al. Potential antidiabetic properties of *Syzygium cumini* (L.) skeels leaf extract-mediated silver nanoparticles. *Austin. J. Anal. Pharm. Chem.* **11**, 1168 (2024).

[45] Ramesh Babu, Y. Does the combined glucose and vitamin c nutritional regimens extend the lifespan of *Drosophila* melanogaster?. *Int. J. Curr. Res.* **11**, 2063–2065 (2019).

[46] Mohammad, F., Singh, P. & Sharma, A. A *Drosophila* systems model of pentylenetetrazole induced locomotor plasticity responsive to antiepileptic drugs. *BMC Syst. Biol.* **3**, 11 (2009). 19154620 10.1186/1752-0509-3-11 PMC2657775

[47] Moghimi, S. & Harini, B. P. A comparative study of the efficiency of *Withania somnifera* and carbamazepine on lifespan, reproduction and epileptic phenotype–a study in *Drosophila* paralytic mutant. *J. Ayurveda Integr. Med.* **13**, 100534 (2022). 34980523 10.1016/j.jaim.2021.11.002 PMC8814379

[48] Linderman, J. A., Chambers, M. C., Gupta, A. S. & Schneider, D. S. Infection-related declines in chill coma recovery and negative geotaxis in *Drosophil*a melanogaster. *PLoS ONE* **7**, e41907 (2012). 23028430 10.1371/journal.pone.0041907 PMC3441536

[49] Gerber, B. et al. Visual learning in individually assayed *Drosophila* larvae. *J. Exp. Biol.* **207**, 179–188 (2004). 14638844 10.1242/jeb.00718

[50] Wong, R., Piper, M. D. W., Wertheim, B. & Partridge, L. Quantification of food intake in *Drosophila*. *PLoS ONE* **4**, e6063 (2009). 19557170 10.1371/journal.pone.0006063 PMC2698149

[51] Dare, S. S. et al. *Drosophila* parabss flies as a screening model for traditional medicine: Anticonvulsant effects of *Annona senegalensis*. *Front. Neurol.* **11**, 606919 (2021). 33519685 10.3389/fneur.2020.606919 PMC7838503

[52] Lodhi, S., Jain, A., Jain, A. P., Pawar, R. S. & Singhai, A. K. Effects of flavonoids from *Martynia annua* and *Tephrosia purpurea* on cutaneous wound healing. *Avicenna J. Phytomed.* **6**, 578–591 (2016). 27761428 PMC5052421

[53] Rana, Z. H., Alam, M. K. & Akhtaruzzaman, M. Nutritional composition, total phenolic content, antioxidant and α-amylase inhibitory activities of different fractions of selected wild edible plants. *Antioxidants* **8**, 203 (2019). 31266183 10.3390/antiox8070203 PMC6680810

[54] Mali, P. C., Ansari, A. S. & Chaturvedi, M. Antifertility effect of chronically administered *Martynia annua* root extract on male rats. *J. Ethnopharmacol.* **82**, 61–67 (2002). 12241978 10.1016/s0378-8741(02)00084-3

[55] Lodhi, S. & Singhai, A. Preliminary pharmacological evaluation of *Martynia annua* Linn leaves for wound healing. *Asian Pac. J. Trop. Biomed.* **1**, 421–427 (2011). 23569806 10.1016/S2221-1691(11)60093-2 PMC3614220

[56] Liu, M. L., Chen, B. B., Li, C. M. & Huang, C. Z. Carbon dots: Synthesis, formation mechanism, fluorescence origin and sensing applications. *Green Chem.* **21**, 449–471 (2019).

[57] Yalshetti, S. et al. Microwave-assisted synthesis, characterization and in vitro biomedical applications of *Hibiscus rosa-sinensis* Linn.-mediated carbon quantum dots. *Sci. Rep.* **14**, 9915 (2024). 38689005 10.1038/s41598-024-60726-y PMC11061284

[58] Shaikh, A. F. et al. Bioinspired carbon quantum dots: An antibiofilm agents. *J. Nanosci. Nanotechnol.* **19**, 2339–2345 (2019). 30486995 10.1166/jnn.2019.16537

[59] Setianto, S., Men, L. K., Bahtiar, A., Panatarani, C. & Joni, I. M. Carbon quantum dots with honeycomb structure: A novel synthesis approach utilizing cigarette smoke precursors. *Sci. Rep.* **14**, 1996 (2024). 38263381 10.1038/s41598-024-52106-3 PMC10806174

[60] Balakrishnan, T., Ang, W. L., Mahmoudi, E., Mohammad, A. W. & Sambudi, N. S. Formation mechanism and application potential of carbon dots synthesized from palm kernel shell via microwave assisted method. *Carbon Resour. Convers.* **5**, 150–166 (2022).

[61] Sharma, N., Sharma, I. & Bera, M. K. Microwave-assisted green synthesis of carbon quantum dots derived from *Calotropis gigantea* as a fluorescent probe for bioimaging. *J. Fluoresc.* **32**, 1039–1049 (2022). 35262854 10.1007/s10895-022-02923-4

[62] Fawaz, W., Hasian, J. & Alghoraibi, I. Synthesis and physicochemical characterization of carbon quantum dots produced from folic acid. *Sci. Rep.* **13**, 18641 (2023). 37903841 10.1038/s41598-023-46084-1 PMC10616078

[63] Ssempijja, F. et al. Attenuation of seizures, cognitive deficits, and brain histopathology by phytochemicals of *Imperata cylindrica (L.) P. Beauv* (Poaceae) in acute and chronic mutant *Drosophila melanogaster* epilepsy models. *J. Evid. Based Complement. Altern. Med.* **28**, 2515690231160191 (2023). 10.1177/2515690X231160191 PMC9989407 36866635

[64] Reynolds, E. R. et al. Treatment with the antiepileptic drugs phenytoin and gabapentin ameliorates seizure and paralysis of *Drosophila* bang-sensitive mutants. *J. Neurobiol.* **58**, 503–513 (2004). 14978727 10.1002/neu.10297

[65] Mishra, P. K. et al. Wood-based cellulose nanofibrils: Haemocompatibility and impact on the development and behaviour of *Drosophila melanogaster*. *Biomolecules* **9**, 363 (2019). 31412664 10.3390/biom9080363 PMC6722666

[66] Priyadarsini, S. et al. Oral administration of graphene oxide nano-sheets induces oxidative stress, genotoxicity, and behavioral teratogenicity in *Drosophila melanogaster*. *Environ. Sci. Pollut. Res.* **26**, 19560–19574 (2019). 10.1007/s11356-019-05357-x 31079296

[67] Singh, M. P. et al. Synthesis of green engineered silver nanoparticles through *Urtica dioica*: An inhibition of microbes and alleviation of cellular and organismal toxicity in *Drosophila melanogaster*. *Antibiotics* **11**, 1690 (2022). 36551347 10.3390/antibiotics11121690 PMC9774676

[68] Shamapari, R. & Nagaraj, K. Teratogenic impacts of Antiepileptic drugs on development, behavior and reproduction in *Drosophila melanogaster*. *Neurotoxicol. Teratol.* **100**, 107305 (2023). 37805079 10.1016/j.ntt.2023.107305

[69] Blum, A. L., Li, W., Cressy, M. & Dubnau, J. Short- and long-term memory in *Drosophila* require cAMP signaling in distinct neuron types. *Curr. Biol.* **19**, 1341–1350 (2009). 19646879 10.1016/j.cub.2009.07.016 PMC2752374

[70] Ploski, J. E. & Aplan, P. D. Characterization of DNA fragmentation events caused by genotoxic and non-genotoxic agents. *Mutat. Res. Fund. Mol. Mech. Mutagen.* **473**, 169–180 (2001). 10.1016/s0027-5107(00)00147-0 11166035

[71] Sui, X. et al. Graphene quantum dots enhance anticancer activity of cisplatin via increasing its cellular and nuclear uptake. *Nanomed. Nanotechnol. Biol. Med.* **12**, 1997–2006 (2016). 10.1016/j.nano.2016.03.010 27085903

[72] Kroll, J. R., Saras, A. & Tanouye, M. A. Drosophila sodium channel mutations: Contributions to seizure-susceptibility. *Exp. Neurol.* **274**, 80–87 (2015). 26093037 10.1016/j.expneurol.2015.06.018 PMC4644469

[73] Edelsparre, A. H., Vesterberg, A., Lim, J. H., Anwari, M. & Fitzpatrick, M. J. Alleles underlying larval foraging behaviour influence adult dispersal in nature. *Ecol. Lett.* **17**, 333–339 (2014). 24386971 10.1111/ele.12234

[74] Chen, K.-F. & Crowther, D. C. Functional genomics in *Drosophila* models of human disease. *Brief. Funct. Genom.* **11**, 405–415 (2012). 10.1093/bfgp/els038 22914042

[75] Okanari, K. et al. Behavioral and neurotransmitter changes on antiepileptic drugs treatment in the zebrafish pentylenetetrazol-induced seizure model. *Behav. Brain Res.* **464**, 114920 (2024). 38403178 10.1016/j.bbr.2024.114920

[76] Tolou-Ghamari, Z., Zare, M., Habibabadi, J. M. & Najafi, M. R. A quick review of carbamazepine pharmacokinetics in epilepsy from 1953 to 2012. *J. Res. Med. Sci.* **18**, S81-85 (2013). 23961295 PMC3743329

